# Carcinogenic effect of potassium octatitanate (POT) fibers in the lung and pleura of male Fischer 344 rats after intrapulmonary administration

**DOI:** 10.1186/s12989-019-0316-2

**Published:** 2019-09-02

**Authors:** Mohamed Abdelgied, Ahmed M. El-Gazzar, William T. Alexander, Takamasa Numano, Masaaki Iigou, Aya Naiki-Ito, Hiroshi Takase, Akihiko Hirose, Yuhji Taquahashi, Jun Kanno, Mona Abdelhamid, Khaled Abbas Abdou, Satoru Takahashi, David B. Alexander, Hiroyuki Tsuda

**Affiliations:** 1 Nanotoxicology Project, Nagoya City University, 3-1 Tanabe-Dohri, Mizuho-ku, Nagoya, 466- 8603 Japan; 2 Department of Experimental Pathology and Tumor Biology, Nagoya City University Graduate School of Medical Sciences, Nagoya, Japan; 3 Department of Forensic Medicine and Toxicology, Faculty of Veterinary Medicine, Beni-Suef University, Beni-Suef, Egypt; 4 Department of Forensic Medicine and Toxicology, Faculty of Veterinary Medicine, Alexandria University, Alexandria, Egypt; 5 Core Laboratory, Nagoya City University Graduate School of Medical Sciences, Nagoya, Japan; 6 Division of Risk Assessment, National Institute of Health Sciences, Kawasaki, Japan; 7 Division of Cellular and Molecular Toxicology, National Institute of Health Sciences, Kawasaki, Japan; 8 Japan Industrial Safety and Health Association, Japan Bioassay Research Center, Kanagawa, Japan; 9 Department of Biochemistry, Nagoya City University Graduate School of Medical Sciences, Nagoya, Japan; 10 Department of Biochemistry, Faculty of Veterinary Medicine, Beni-Suef University, Beni-Suef, Egypt

**Keywords:** Potassium octatitanate fibers, MWCNT-7, Lung carcinoma, Malignant mesothelioma, Intra-tracheal intra-pulmonary spraying, TIPS

## Abstract

**Background:**

Potassium octatitanate fibers (K_2_O•8TiO_2_, POT fibers) are used as an asbestos substitute. Their physical characteristics suggest that respirable POT fibers are likely to be carcinogenic in the lung and pleura. However, previous 2-year inhalation studies reported that respired POT fibers had little or no carcinogenic potential. In the present study ten-week old male F344 rats were left untreated or were administered vehicle, 0.25 or 0.5 mg rutile-type nano TiO_2_ (r-nTiO_2_), 0.25 or 0.5 mg POT fibers, or 0.5 mg MWCNT-7 by intra-tracheal intra-pulmonary spraying (TIPS), and then observed for 2 years.

**Results:**

There were no differences between the r-nTiO_2_ and control groups. The incidence of bronchiolo-alveolar cell hyperplasia was significantly increased in the groups treated with 0.50 mg POT and 0.50 mg MWCNT-7. The overall incidence of lung tumors, however, was not increased in either the POT or MWCNT-7 treated groups. Notably, the carcinomas that developed in the POT and MWCNT-7 treated rats were accompanied by proliferative fibrous connective tissue while the carcinomas that developed in the untreated rats and the r-nTiO_2_ treated rats were not (carcinomas did not develop in the vehicle control rats). In addition, the carcinoma that developed in the rat treated with 0.25 mg POT was a squamous cell carcinoma, a tumor that develops spontaneously in about 1 per 1700 rats. The incidence of mesothelial cell hyperplasia was 4/17, 7/16, and 10/14 and the incidence of malignant mesothelioma was 3/17, 1/16, and 2/14 in the 0.25 mg POT, 0.5 mg POT, and MWCNT-7 treated groups, respectively. Neither mesothelial cell hyperplasia nor mesothelioma developed in control rats or the rats treated with r-nTiO_2_. Since the incidence of spontaneously occurring malignant mesothelioma in rats is extremely low, approximately 1 per 1000 animals (Japan Bioassay Research Center [JBRC] historical control data), the development of multiple malignant mesotheliomas in the POT and MWCNT-7 treated groups was biologically significant.

**Conclusion:**

The incidence of pleural mesotheliomas in male F344 rats administered POT fibers and MWCNT-7 was significantly higher than the JBRC historical control data, indicating that the incidence of pleural mesothelioma in the groups administered POT fibers and MWCNT-7 fibers via the airway using TIPS was biologically significant. The incidence of type II epithelial cell hyperplasia and the histology of the carcinomas that developed in the POT treated rats also indicates that respirable POT fibers are highly likely to be carcinogenic in the lungs of male F344 rats.

**Electronic supplementary material:**

The online version of this article (10.1186/s12989-019-0316-2) contains supplementary material, which is available to authorized users.

## Background

Potassium octatitanate fibers (K_2_O•8TiO_2_, POT fibers) are used as an asbestos substitute. This material is heat resistant and chemically stable. Their long thin shape allows respired POT fibers to be deposited beyond the ciliated airways and their stability makes them biopersistent: POT fibers have been recovered intact from the rat lung 1 year after inhalation [1]. The fiber pathogenicity paradigm identifies three characteristics of inhaled fibers that affect their pathogenicity: an aerodynamic diameter that allows deposition beyond the ciliated airways, sufficient length and rigidity to impede phagocytosis and removal of the fiber by macrophages, and a composition that allows the fiber to retains its structural integrity [2]. Thus, respired POT fibers are identified by the fiber pathogenicity paradigm as potentially carcinogenic.

Initial studies of POT fibers applied 40 mg of the material directly to the pleural surface of female Osborne-Mendel rats and reported development of pleural tumors [3, 4]. An inhalation study exposed groups of hamsters, guinea pigs, and rats to 39, 79, 82, and 371 mg/m^3^ POT fibers for 3 months and followed the animals for an additional 15 to 24 months [5]. In this study, one hamster in each of the 79, 82, and 371 mg/m^3^ groups developed pleural mesothelioma, but none of the exposed guinea pigs or rats developed pleural tumors, and none of the exposed groups exhibited an increase in lung tumors compared to the controls. A later intraperitoneal injection study reported that injection of 5 and 10 mg POT fibers resulted in the development of peritoneal mesotheliomas [6]. Based in part on the results of these studies and the characteristic biopersistence of POT fibers, the WHO Workshop on Mechanisms of Fibre Carcinogenesis and Assessment of Chrysotile Asbestos Substitutes issued a statement that “respirable potassium octatitanate fibres are likely to pose a high hazard to humans after inhalation exposure” [7].

The studies cited above, however, used extremely high amounts of POT fibers. None of the more recent studies of POT fibers report POT-induced development of tumors. While most of these studies followed the animals for 1 year or less, three inhalation studies that followed the animals for up to 2 years have been reported [8–10]. Taken together, the studies on POT fibers indicate that these fibers can be carcinogenic, but that very high amounts of fibers in prolonged contact with susceptible tissues are required to induce carcinogenesis and that inhalation exposure may not be carcinogenic. [See Additional file 1 for a brief discussion of in vivo POT fiber studies.]

Thus, the physical characteristics of POT fibers suggest that they are potentially carcinogenic, but animal studies indicate that POT fibers have little carcinogenic potential. One possible reason for this apparent discrepancy is the titanium dioxide make-up of the fibers. Therefore, we initially performed a short-term experiment comparing the lung toxicity of two types of non-fibrous titanium dioxide nanoparticles, rutile (r-nTiO_2_) and anatase (a-nTiO_2_), with that of POT fibers [11]. We administered r-nTiO_2_, a-nTiO_2_, and POT fibers to rats via the airway using intra-tracheal intra-pulmonary spraying (TIPS) and found that like other materials, the biopersistence and toxicity of the fiber was greater than that of the non-fibrous nano-particles. Thus, the titanium dioxide composition of POT fibers does not appear to explain their lack of carcinogenicity. Therefore, we conducted the present 2-year study comparing the lung toxicity of TIPS-administered POT fibers with that of r-nTiO_2_ and MWCNT-7, a known lung carcinogen in rats.

## Results

### Characterization of the test materials

Transmission electron microscopic (TEM) images of r-nTiO_2_, POT fibers, and MWCNT-7 fibers are shown in Additional file 2: Figure S1: derived from Abdelgied et al., 2019 [12]. r-nTiO_2_ forms large agglomerates composed of numerous particles while POT and MWCNT-7 fibers are mainly present as single fibers or small loose agglomerates: agglomerate is used as defined in Nichols et al. 2002 [13]. POT fibers have relatively thick straight shapes and MWCNT-7 fibers are thin and more flexible. The mean length and width of individual r-nTiO_2_ particles was 50.02 ± 8.24 nm and 14.35 ± 4.63 nm, respectively; the mean length and width of the POT fibers was 6.06 ± 1.53 μm and 305 ± 69 nm, respectively (Additional file 2: Figure S2; derived from Abdelgied et al., 2018 [11]); and the mean length and width of the MWCNT-7 fibers was 5.31 ± 3.81 μm and 75.65 ± 20.54 μm, respectively [11, 12]. There were no discernable changes in the size distribution of the test materials after aerosolization with the microsprayer. Element analysis showed that r-nTiO_2_ nanoparticles were composed of 60% titanium and 40% oxygen, and POT fibers were composed of 52.1% titanium, 37.0% oxygen, and 10.8% potassium, in agreement with the molecular ratio of each element in these materials.

### Body weights, clinical signs, and survival

The disposition and survival of the animals used in this study is summarized in Additional file 3: Table S1 and Additional file 4: Figure S1. There was a significant decrease in survival of the rats administered MWCNT-7. There were no differences in survival rates between any of the r-nTiO_2_ or POT treated groups and the vehicle control group.

The first treatment-related death occurred on day 507 (week 73): one rat in the MWCNT-7 group died due to a bronchiolo-alveolar adenocarcinoma. Therefore, all rats surviving for 73 weeks were included in the assessment of r-nTiO_2_, POT, and MWCNT-7 toxicity/carcinogenicity. Prior to week 73, 16 rats died from non-treatment related causes (Additional file 3: Table S2) and were excluded from toxicity/carcinogenicity assessment. Body weight curves are shown in Additional file 4: Figure S2. There was a slight, but statistically significant, reduction in the body weights of the rats in the vehicle control group compared to the untreated group at week 105 (Additional file 3: Table S3). There were no differences between the body weights of the rats in the treated groups compared to the vehicle control group (Additional file 3: Table S3 and Additional file 4: Figure S2).

No treatment-related deaths or clinical signs were observed in either of the two r-nTiO_2_ treated groups. Seven treatment-related deaths occurred prior to the end of the study in the POT and MWCNT-7 groups (Additional file 3: Table S4). In the 0.25 mg POT group, one rat had malignant visceral mesothelioma and a bronchiolo-alveolar adenoma (sacrificed wk. 94) and a second rat had malignant parietal mesothelioma (sacrificed wk. 98). In the 0.50 mg POT group, one rat had malignant parietal mesothelioma and a bronchiolo-alveolar adenocarcinoma (died wk. 75). In the MWCNT-7 group, one rat had a bronchiolo-alveolar adenocarcinoma (died wk. 73), a second rat had malignant parietal mesothelioma and a bronchiolo-alveolar carcinoma (sacrificed wk. 91), a third rat had a bronchiolo-alveolar adenocarcinoma (died wk. 92), and a fourth rat had malignant parietal mesothelioma (died wk. 92).

### Macroscopic findings and organ weights

**Fig. 1:**
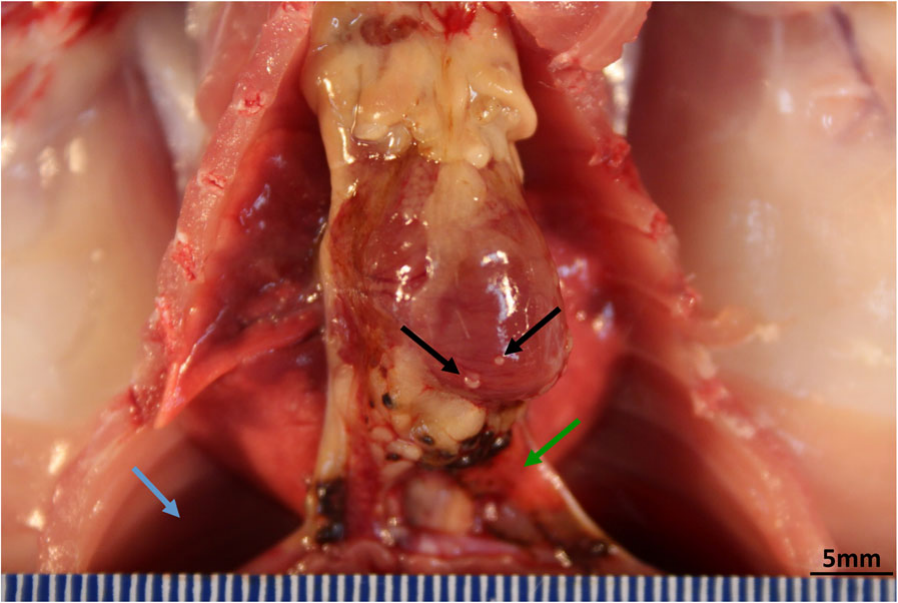
Gross appearance of a malignant mesothelioma from a rat in the 0.25 mg POT fiber treated group (died at 98 week) showing small nodules on the surface of the pericardium (black arrows) and red pleural effusion in the thoracic cavity. In this animal, the mediastinal area of the lung was adherent to the diaphragm (green arrow)

Multiple grayish white nodules were found in the lungs of some of the rats in the untreated and vehicle control groups, as well as the treated groups, and were therefore concluded to be unrelated to treatment. The lungs of the MWCNT-7 treated rats tended to be dark grayish in color. In the 0.25 mg POT fiber treated group, one animal had small whitish nodules on the surface of the pericardium and adhesion of the mediastinal area of the lung to the diaphragm with bloody fluid in the thoracic cavity (Fig. 1), and two other animals showed whitish nodules on the inner surface of the thoracic cavity and diaphragm. In the 0.50 mg POT fiber treated group, one animal had white masses on the lung and adhesion of the lung to the thoracic wall. In the MWCNT-7 treated group, two animals had white nodules on the paravertebral inner aspect of the thoracic wall. A few tumor masses were found in other organs of some of the experimental animals of the untreated, vehicle control, and treated groups (Additional file 3: Table S5). These tumors were unrelated to treatment.

Absolute and relative organ weights are shown in Additional file 3: Tables S6 and S7. The absolute and relative lung weights were significantly increased in the MWCNT-7 treated rats. The relative but not absolute brain, heart, liver, and kidney weights were significantly increased in MWCNT-7 treated rats. There were no differences in the absolute or relative weights of any organs in the r-nTiO_2_ or POT treated groups compared to the vehicle control group.

### Microscopic findings

**Fig. 2:**
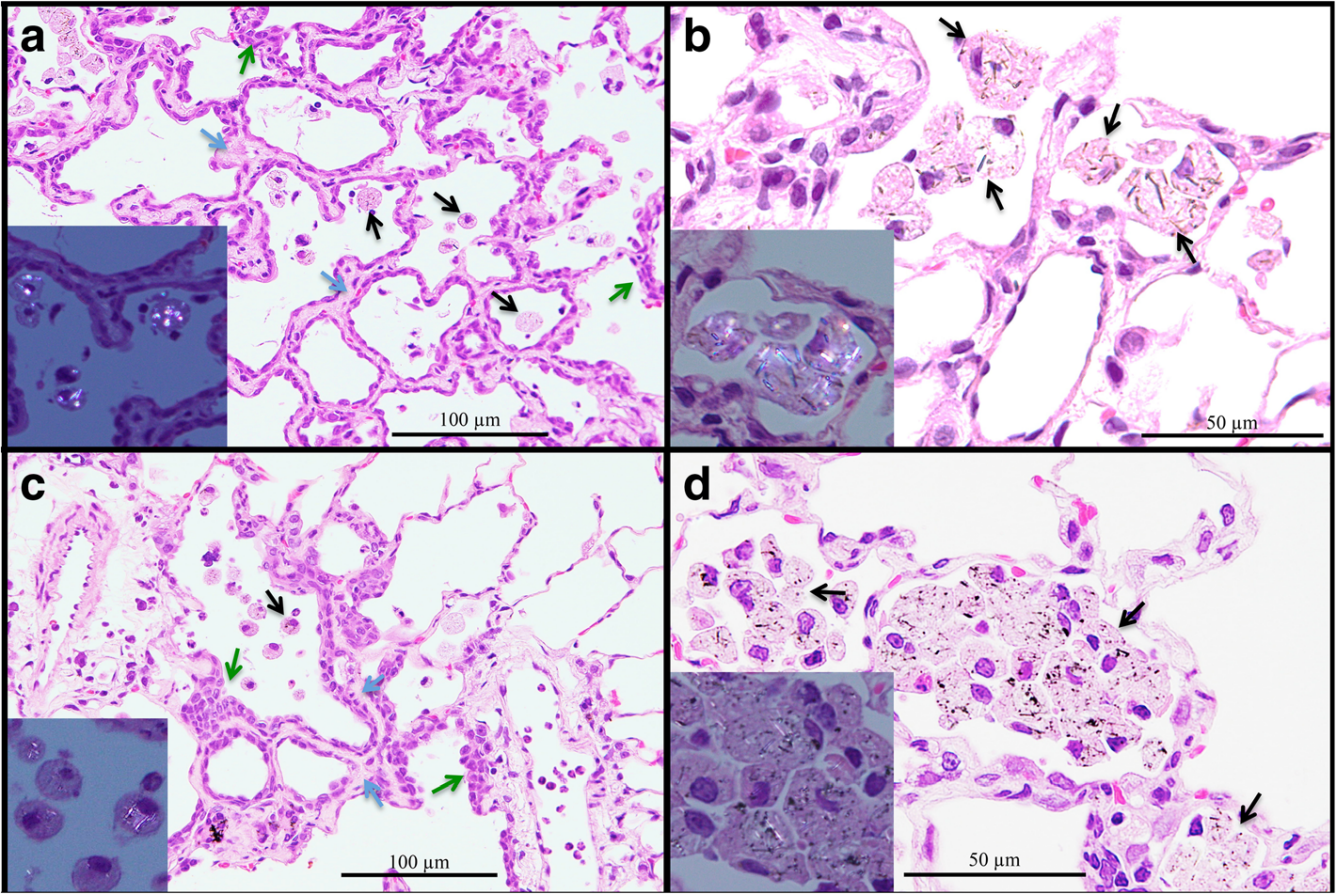
Pulmonary alveolar cell hyperplasia in rats in the 0.50 mg POT (**a**, **b**) and 0.50 mg MWCNT-7 (**c**, **d**) treated groups. Infiltration of alveolar macrophages phagocytizing POT fibers and MWCNT-7 (black arrows), thickening and fibrosis of the alveolar wall (blue arrows), and hyperplasia of alveolar epithelium (green arrows) can be seen. Insert, polarized light microscope images

**Fig. 3:**
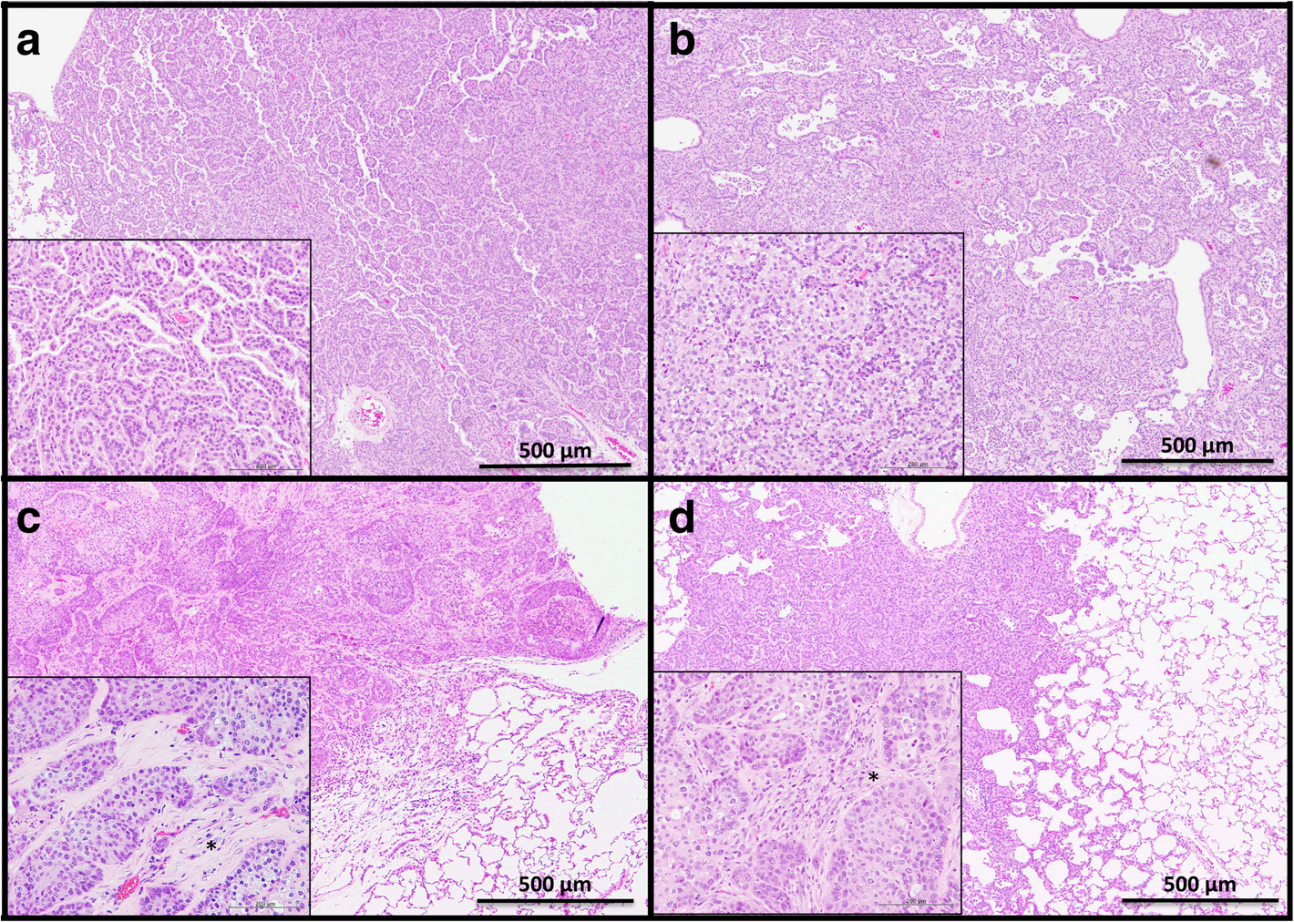
Pulmonary bronchiolo-alveolar cell carcinomas from rats in the untreated (**a**), 0.50 mg r-nTiO_2_ (**b**), 0.50 mg POT (**c**), and 0.50 mg MWCNT**-**7 (**d**) treated groups. Proliferative fibrous connective tissue can be seen in the carcinomas from the rats in the POT and MWCNT-7 treated groups (asterisks in panel c and d inserts), but not in the carcinomas from the rats in the untreated and r-nTiO_2_ treated groups

**Fig. 4:**
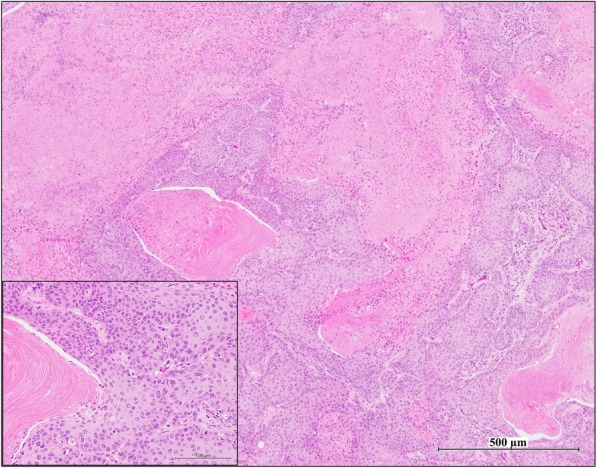
Squamous cell carcinoma from a rat in the 0.25 mg POT treated group

Proliferative lesions of the lung and the pleura are shown in Table 1. The incidence of type II alveolar cell hyperplasia was significantly increased in the 0.50 mg POT and MWCNT-7 treated groups compared to the vehicle control group (Fig. 2). Lung tumor incidence was not increased in either the POT or MWCNT-7 treated groups. Importantly, however, the bronchiolo-alveolar carcinomas that developed in the POT and MWCNT-7 treated rats was accompanied by proliferative fibrous connective tissue, while the spontaneous bronchiolo-alveolar carcinomas that developed in the untreated controls, as well as the bronchiolo-alveolar carcinomas that developed in the r-nTiO_2_ treated rats, did not have proliferative fibrous connective tissue (Fig. 3): bronchiolo-alveolar carcinomas did not develop in the vehicle control rats. In addition, one rat in the 0.25 mg POT group developed a squamous cell carcinoma (Fig. 4). While development of a single tumor does not have statistical significance, the incidence of the spontaneous development of squamous cell carcinoma in the historical control data of the Japan Bioassay Research Center is 2/3398 in male rats and 0/3197 in female rats. Consequently, the histology of the carcinomas that developed in the lungs of the POT and MWCNT-7 treated rats indicates that these tumors are very probably treatment related.

**Table 1:** Proliferative lesions in the lung and pleura

Group	Untreated	Vehicle	r-nTiO_2_ 0.25 mg	r-nTiO_2_ 0.50 mg	POT 0.25 mg	POT 0.50 mg	MWCNT-7 0.50 mg
Number of animals	19	19	20	19	17	16	14
Bronchiolo-alveolar hyperplasia	2	2	0	2	7	10**	14^a,^***
Bronchiolo-alveolar adenoma	0	1	2	0	1	0	1
Bronchiolo-alveolar adenocarcinoma	3	0	0	2	0	2	5
Squamous cell carcinoma	0	0	0	0	1	0	0
Mesothelial cell hyperplasia	0	0	0	0	4*	7**	10***
Visceral mesothelioma	0	0	0	0	1	0	0
Parietal mesothelioma	0	0	0	0	2	1	2
Carcinoma + Mesothelioma	3	0	0	2	4	2^b^	6^b^

^a^Three animals also had bronchiolar hyperplasia

^b^One animal had bronchiolo-alveolar carcinoma and parietal mesothelioma

*^,^**^,^***Different from both the Untreated and the Vehicle Control at *p* < 0.05, *p* < 0.01, *p* < 0.001, respectively

**Fig. 5:**
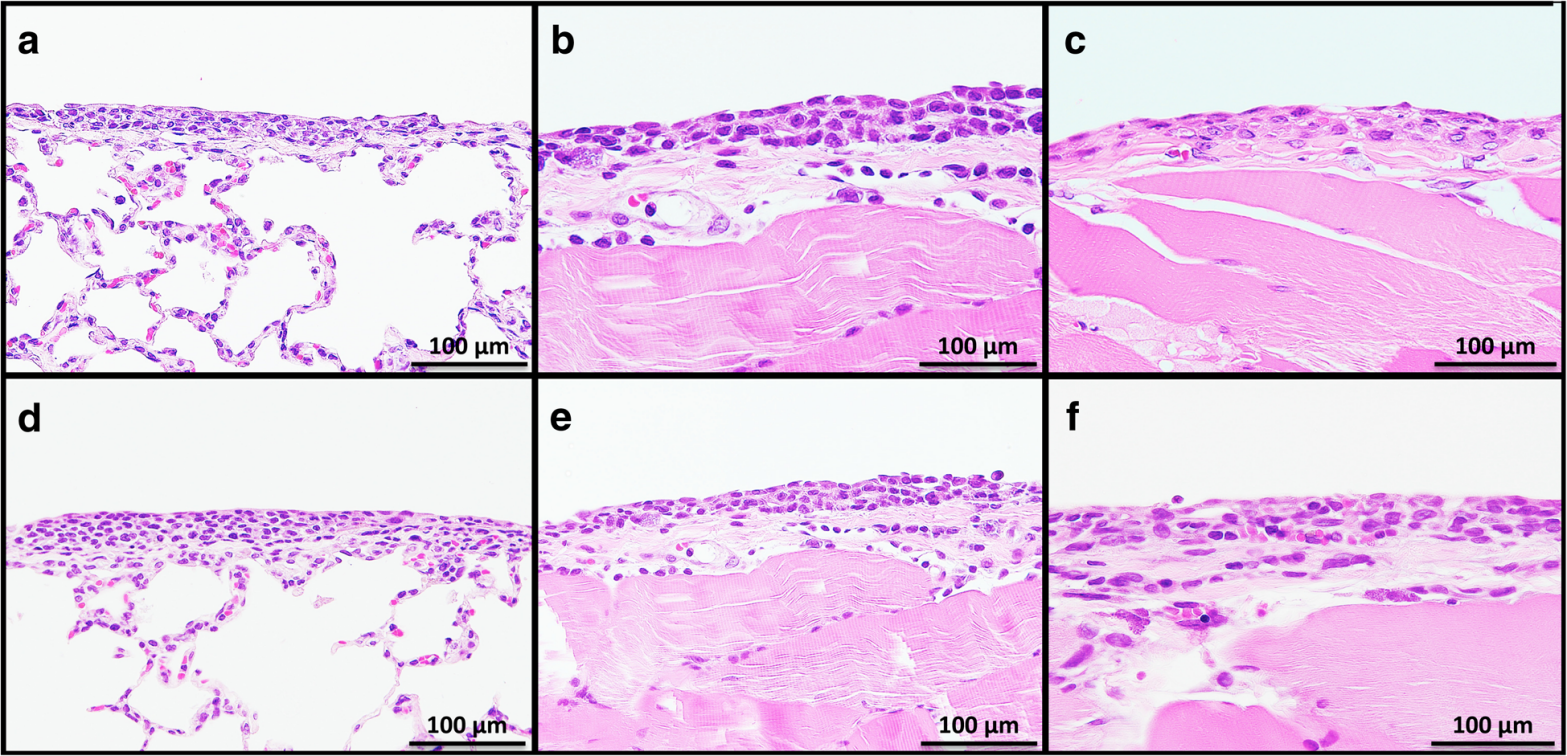
Pleural hyperplasia in rats in the POT fiber (**a**, **b**, **c**) and MWCNT-7 (**d**, **e**, **f**) treated groups. Visceral pleura (**a**, **d**), diaphragmatic region of the parietal pleura (**b**, **e**), and costal region of the parietal pleura (**c**, **f**)

**Fig. 6:**
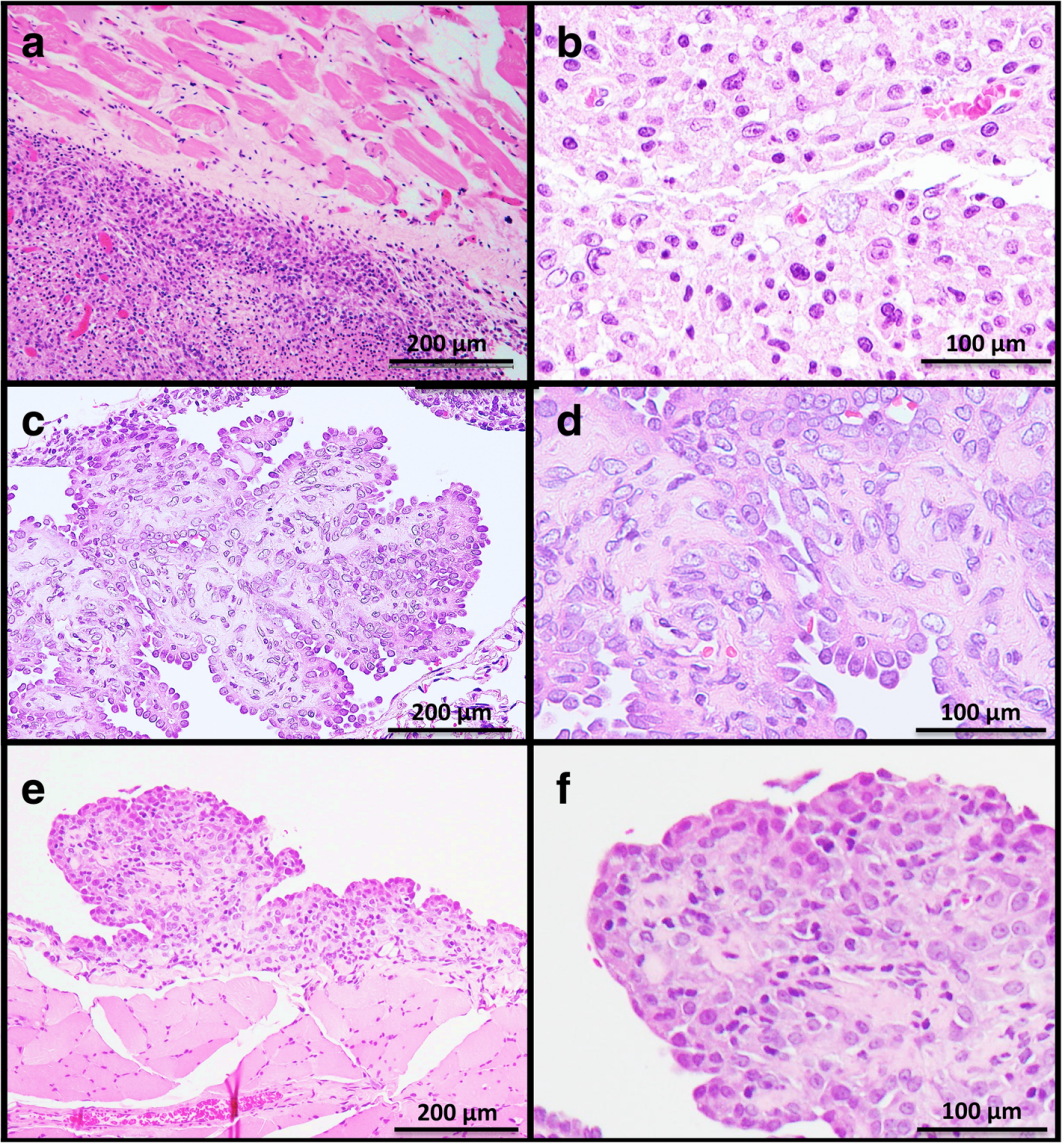
Malignant pleural mesotheliomas. A sarcomatoid type mesothelioma of the costal region of the parietal pleura in a rat from the 0.50 mg POT fiber treated group (**a**, **b**). An epitheliod type mesothelioma of the diaphragmatic region of the parietal pleura invading into the pleural cavity and the visceral pleura in a rat from the 0.25 mg POT treated group (**c**, **d**). An epitheliod type mesothelioma of the costal region of the parietal pleura in a rat from the MWCNT-7 group (**e**, **f**)

The incidences of mesothelial cell hyperplasia (Fig. 5) were significantly increased in the POT and MWCNT-7 treated groups compared to the vehicle controls. The incidence of malignant mesothelioma was not significantly increased in the POT or MWCNT-7 treated groups. Two rats in 0.25 mg POT group, one rat in the 0.50 mg POT group, and two rats in the MWCNT-7 group developed sarcomatoid type malignant pleural mesotheliomas and one rat in the 0.25 mg POT group developed an epitheliod type malignant pleural mesothelioma (Fig. 6). No animals in the untreated, vehicle control, or r-nTiO2 groups developed either mesothelial cell hyperplasia or mesothelioma. Importantly, the incidence of spontaneous development of malignant mesothelioma in the historical control data of the Japan Bioassay Research Center is 3/3398 male rats. Consequently, development of multiple mesotheliomas in POT and MWCNT-7 treated rats indicates that these tumors are treatment-related (also see the Preamble to the IARC Monographs [amended January 2019] [14]).

Both POT and MWCNT-7 fibers were detected in the lungs, trachea, mediastinal lymph nodes, kidney, spleen, liver, and testis (data not shown). In contrast, and unlike the 1-year sacrifice (see Additional file 4: Figure S3 in Abdelgied et al., 2019 [12]), r-nTiO2 particles were not detected in any of these organs. Alveolar macrophage numbers were increased in the POT and MWCNT-7 treated rats (Fig. 2, Additional file 3: Table S8). Focal fibrosis, characterized by increased collagen deposition and thickening of the alveolar wall, was also increased in the POT and MWCNT-7 treated rats, and the incidence of pulmonary granulomatous inflammation was increased in MWCNT-7 treated rats (Fig. 2 and Additional file 3: Table S8).

Neither POT nor MWCNT-7 fibers were detected in the tissue sections prepared for histopathological examination that contained mesothelial hyperplasia or mesothelioma.

### Observation of test materials in the lung, mediastinal lymph nodes, and pleural cavity lavage fluid

**Fig. 7:**
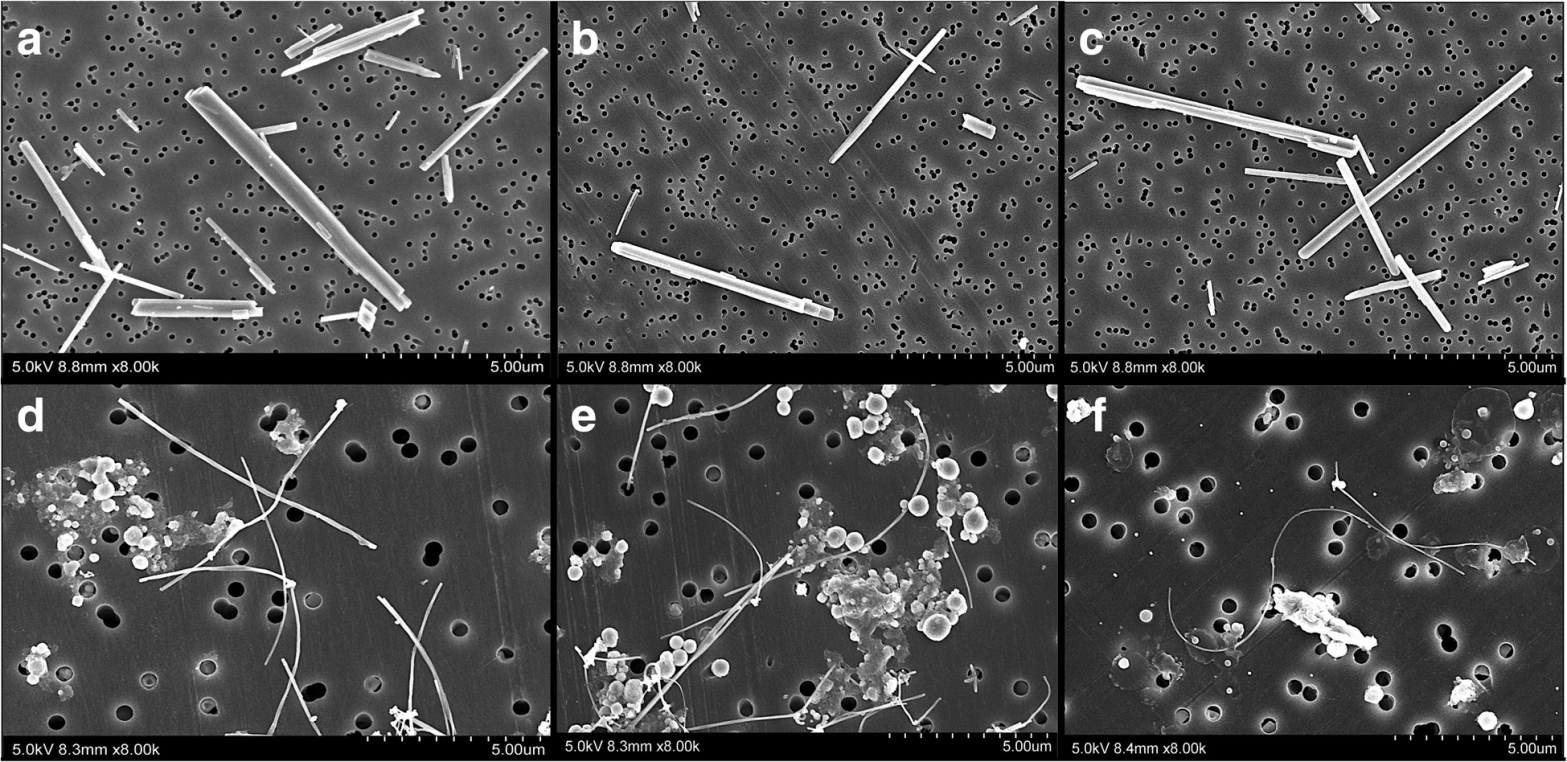
SEM of POT fibers (**a**, **b**, **c**) and MWCNT-7 (**d**, **e**, **f**) in digested lung (**a**, **d**) and mediastinal lymph nodes (**b**, **e**), and in the pleural cavity lavage cell pellet (**c**, **f**)

Table 2 shows the lengths and widths of POT and MWCNT-7 fibers obtained from digested lung tissue, mediastinal lymph nodes, and the pleural cavity lavage sample. r-nTiO2 particles could not be detected in the lung, mediastinal lymph nodes, or pleural cavity lavage samples. Figure 7 panels a, b, d, and e show SEM images of POT and MWCNT-7 fibers obtained from digested lung and mediastinal lymph node tissue specimens. The POT fibers from the lung were 8.13 ± 6.90 μm in length and 329.90 ± 173.22 nm in width, and the POT fibers from the mediastinal lymph nodes were 4.95 ± 2.57 μm in length and 297.75 ± 104.85 nm in width. The MWCNT-7 fibers from the lung were 4.48 ± 2.54 μm in length and 90.18 ± 31.84 nm in width, and the MWCNT-7 fibers from the mediastinal lymph nodes were 5.83 ± 1.54 μm in length and 39.54 ± 5.90 nm in width. The amounts of POT fibers detected in the mediastinal lymph nodes were lower than the amount of MWCNT-7 (Additional file 4: Figure S3).

**Table 2:** Size of test materials in suspension before administration and materials collected from the lung, mediastinal lymph node, and pleural cavity cell pellet at 2 years

		r-nTiO_2_	POT	MWCNT-7
Suspension	Length (μm)	0.050 ± 0.008	6.06 ± 1.53	5.31 ± 3.81
	Width (nm)	14.35 ± 4.63	305.00 ± 69.00	75.65 ± 20.54
Lung	Length (μm)	ND	8.13 ± 6.90	4.48 ± 2.54
	Width (nm)	ND	329.90 ± 173.22	90.18 ± 31.84
Mediastinal lymph node	Length (μm)	ND	4.95 ± 2.57	5.83 ± 1.54
	Width (nm)	ND	297.75 ± 104.85	39.54 ± 5.90
Pleural cavity	Length (μm)	ND	8.93 ± 1.38	10.38 ± 2.76
	Width (nm)	ND	375.73 ± 59.73	42.50 ± 17.02

*ND* Not Detected

Figure 7 panels c and f show SEM images of POT and MWCNT-7 fibers obtained from the pleural cavity lavage sample. The sample from the 0.25 mg POT treated rats contained 570 POT fibers and the sample from the 0.50 mg treated rats contained 650 fibers. POT fibers were 8.93 ± 1.38 μm in length and 375.73 ± 59.73 nm in width. The sample from the MWCNT-7 contained 240 fibers. MWCNT-7 fibers were 10.38 ± 2.76 μm in length and 42.50 ± 17.02 nm in width.

### Lactate dehydrogenase (LDH) activity and total protein (TP) concentration in pleural cavity lavage supernatant

At week 105, LDH activity, an indicator of cellular toxicity, and TP, an indicator of tissue integrity, in the pleural cavity lavage fluid (PLF) didn’t show any significant change in any of the treated groups compared to the controls. However, of the five rats randomly chosen from each group that were used for collection of PLF, 2 rats had bronchiolo-adenomas, 2 rats had bronchiolo-carcinomas, 1 rat had squamous cell carcinoma, and 1 rat had mesothelioma: LDH activity and TP concentration were elevated in these rats (Additional file 3: Table S9).

## Discussion

The dose of POT fibers and nanoparticles used in our initial short-term study was 1.0 mg per rat [11]. This dose was based in part on studies with poorly soluble materials, including POT fibers, showing that lung burdens above approximately 1–3 mg of particles per gram lung tissue can alter retention kinetics [9, 15]: TIPS administration of an initial dose of 1 mg per rat in our short-term study resulted in a lung burden of approximately 1 mg of test material per gram of lung tissue. Another consideration was that in the 2-year inhalation studies by Yamato et al. [10] and Oyabu et al. [9], during the 1 year of inhalation exposure the rats inhaled approximately 30 mg of fibers and retained approximately 2.4 mg of fibers in their lungs. One year after the end of inhalation exposure, the lung burden was approximately 1.4 mg per rat [9]. These levels of fibers were not carcinogenic in these studies, although the study by Yamato et al. (2003) did report a non-significant induction of non-malignant lesions, 3 adenomas and 1 squamous metaplasia, in POT exposed rats [10]. However, the 2-year studies by Ikegami et al. [8], which used approximately twice the concentration of fibers as Yamato et al. (2003) and a 2 year inhalation period, and Oyabu et al. [9], which used approximately the same concentration of fibers and the same 1 year inhalation period as Yamato et al. (2003), reported that inhalation exposure to POT fibers did not result in any proliferative lesions. Therefore, administration of 1 mg per rat should not induce non-specific toxicity. In our short-term study, TIPS administration of 1 mg POT fibers induced tissue responses consistent with these fibers being potential carcinogens (see Abdelgied et al. 2018 [11]). Reasoning that longer exposure to lower levels of fibers should produce similar results to those of our short-term study, in the current study rats were administered final total doses of 0.25 and 0.50 mg r-nTiO_2_, 0.25 and 0.50 mg POT fibers, and 0.50 mg MWCNT-7 fibers over the course of 2 weeks by intra-tracheal intra-pulmonary spraying (TIPS).

There was a significant increase in pleural mesothelial cell hyperplasia and a non-significant increase in pleural mesotheliomas in rats treated with 0.25 mg and 0.50 mg POT fibers and 0.50 mg MWCNT-7 fibers; the untreated, vehicle control, and r-nTiO_2_ treated rats did not develop either mesothelial cell hyperplasia or mesotheliomas (see Table 1). Four of 33 rats administered POT fibers (0.25 mg and 0.50 mg groups combined) and 2 of 14 rats administered MWCNT-7 fibers via TIPS developed pleural mesothelioma. While these incidences are not statistically higher than the controls (Untreated 0/19; Vehicle Control 0/19), the JBRC historical control data shows that only 3 of 3398 rats spontaneously developed malignant pleural mesothelioma. Therefore, based on the JBRC historical control data, development of multiple pleural mesotheliomas in the POT and MWCNT-7 groups was biologically significant (also see the Preamble to the IARC Monographs [amended January 2019] [14]).

We also observed a significant induction of type II epithelial cell hyperplasia in the 0.50 mg POT and MWCNT-7 groups; however, the development of lung tumors was not increased in these groups (see Table 1). Importantly, however, the carcinomas that developed in the POT and MWCNT-7 exposed rats was accompanied by proliferative fibrous connective tissue, while the carcinomas that developed in the untreated and r-nTiO2 treated rats (carcinomas did not develop in any of the vehicle control rats) did not contain fibrous connective tissue. These results are similar to those from the recent 2-year MWCNT-7 inhalation study by Kasai et al. [16] and suggest that the carcinomas that developed in the POT and MWCNT-7 treated rats were not spontaneous, but rather, were treatment-related. In addition, the carcinoma that developed in the rat administered 0.25 mg POT fibers was a squamous carcinoma. While the development of a single tumor does not have statistical significance, the incidence of spontaneous development of squamous cell carcinoma in the historical control data of the Japan Bioassay Research Center is 2/3398 in male rats and 0/3197 in female rats, suggesting that development of this tumor was treatment-related. Overall, the incidence of type II epithelial cell hyperplasia and the histology of the tumors that developed in the r-nTiO2 treated rats was not different from the control groups while the incidence of type II epithelial cell hyperplasia and the histology of the tumors that developed in the POT and MWCNT-7 treated groups indicate that POT fibers are highly likely to be carcinogenic in the rat lung.

Initial studies using POT fibers reported that these fibers can be carcinogenic when in contact with susceptible tissues [3–6]. However, these studies used extremely high amounts of POT fibers, and none of the more recent studies using POT fibers report POT-induced tumor development. One study did report the development of 3 adenomas in 16 rats exposed to POT fibers by inhalation [10]. However, the incidence was not statistically significant compared to the control group (0/16), and no carcinomas developed in the POT exposed rats. Overall, in most previous studies, exposure to POT fibers did not result in tumor development; tumors developed only when susceptible tissues were exposed to excessively high levels of fibers for a prolonged period of time. (See Additional file 1 for a brief discussion of in vivo POT fiber studies.)

Inhaled thin, long biopersistent fibers, such as POT fibers, can be deposited beyond the ciliated airways and are identified by the fiber pathogenicity paradigm as potentially carcinogenic [2]. One factor that could be involved in the apparent discrepancy in the carcinogenicity of POT fibers predicted by the fiber pathogenicity paradigm and the carcinogenicity of the fibers in experimental animal studies is the titanium dioxide makeup of the fibers. Other factors include nasal filtering of the fibers in inhalation studies and aggregation/dispersion of the test material: Yokohira et al. infused POT fibers directly into the pleural cavity of rats and mice and followed the animals for 12–15 months, but while atypical proliferation of pleural mesothelial cells was observed, mesothelioma did not develop in the exposed animals [17, 18]. The authors suggest that one reason for the lack of mesothelioma development was possible aggregation of the fibers in the pleural cavity [18]. Another important factor is the length of time of exposure to carcinogenic levels of POT fibers. In TIPS administration studies the test material is administered at the beginning of the study, and if the material is biopersistent (a crucial property of respired carcinogenic fibers) processes involved in carcinogenesis, including movement of the material from the lung into the pleural cavity and accumulation of the material in the pleural cavity, proceed for the entire study period. In contrast, in inhalation studies a substantial fraction of the study period may be required for sufficient material to accumulate to initiate processes involved in carcinogenesis, leaving insufficient time for tumors to develop.

Since one possible reason for the apparent discrepancy in the carcinogenicity of POT fibers predicted by the fiber pathogenicity paradigm and the carcinogenicity of the fibers in experimental animal studies is the state of aggregation of the fibers, in both the previous short-term study [11] and the present study we used POT fibers prepared using a novel method of dispersion, the Taquann method [19]. The Taquann method suspends the test material in tert-Butanol and then filters the suspension to remove large aggregates. The filtered suspension is then snap-frozen. As explained in the Methods section, shortly before administration, the tert-Butanol was removed and the dispersed fibers were resuspended in vehicle and sonicated. Characterization of the resuspended fibers showed that the fibers were well dispersed and that the suspension did not contain large aggregates.

Our initial short-term study compared the toxicity of POT fibers with the toxicities of titanium dioxide nanoparticles, rutile titanium dioxide nanoparticles (r-nTiO_2_) and anatase titanium dioxide nanoparticles (a-nTiO_2_): all three materials were prepared using the Taquann method and administered using TIPS. We found that like other materials, the biopersistence and toxicity of POT fibers was greater than that of non-fibrous nano-particles of the same chemical makeup [11]. Thus, the titanium dioxide composition of POT fibers does not appear to explain their lack of carcinogenicity.

In the present study we administered Taquann-prepared test materials to rats using TIPS and observed the animals for 2 years: Taquann preparation of the test materials ensures that the materials were well dispersed and that the administered suspension did not contain large aggregates; TIPS administration bypasses the nasal passages of the test animals, eliminating nasal filtering of the test material; TIPS administration also allows processes involved in carcinogenesis to proceed for the entire 2 year experimental period. In our study, we observed a biologically significant induction of malignant pleural mesotheliomas.

As noted above, mesothelioma development was generally not observed in previous studies. One of the primary differences between our study and previous studies is the length of time of exposure to POT fibers. As can be seen in Additional file 1, most of the studies followed the exposed animals for 12–15 months or less, and none of these studies reported development of mesothelioma in the exposed animals. Similarly, in our study, no tumors were observed in the rats sacrificed at 1 year [12]. Only 6 studies followed the exposed animals for 2 years. Of these 6 studies, 1 study exposed animals by intrapleural implantation, 1 study exposed animals by intraperitoneal injection, and 4 studies exposed animals by inhalation. Both the intrapleural implantation and intraperitoneal injection studies reported that POT fibers caused mesotheliomas; although, both of these studies exposed animals to exceedingly high levels of fibers. The 4 two-year inhalation studies reported that exposure to POT fibers did not cause mesotheliomas in rats. As noted above, exposure of the pleural mesothelium to fibers is considerably longer in 2-year instillation studies compared to 2-year inhalation studies. Thus, mesothelioma was only induced in rats in studies that exposed the pleural mesothelium to POT fibers for longer periods of time.

The incidence of type II epithelial cell hyperplasia and the histology of the carcinomas that developed in the POT and MWCNT-7 treated rats indicates that POT and MWCNT-7 fibers are highly likely to be carcinogenic in the lung of male rats. As with induction of mesotheliomas, one of the primary differences between the current study and the previous instillation studies with POT fibers is the length of time that the lung was exposed to high levels of POT fibers: none of the instillation studies followed the test animals for 2 years (see Additional file 1). Most inhalation studies also followed the test animals for less than 2 years. Only four studies that exposed the lung to POT fibers followed the test animals for 2 years; these were all inhalation studies. Ikegami et al. (2004) exposed male F344 rats to up to 200 WHO POT fibers/cm^3^ for 6 h/d, 5 d/wk. for 2 years [8]. At the end of the 2-yr exposure period the fiber burden in the lung was approximately 520 fibers/μg dry weight (calculation of the fiber burden is shown in Additional file 5). Yamato et al. (2003) exposed male Wistar rats to 111 POT fibers/cm^3^ for 6 h/d, 5 d/wk. for 1 year [10]. At the end of the 1-yr exposure period the fiber burden in the lung was approximately 361 fibers/μg dry weight. Oyabu et al. (2004) exposed male Wistar rats to POT fibers for 6 h/d, 5 d/wk. for 1 year (fiber number per unit volume was not stated) [9]. At the end of the 1-yr exposure period the fiber number burden in the lung was not stated, however, the amount of POT fibers that accumulated in the lung was 2.39 ± 0.50 mg/lung, which was similar to the lung burden reported by Yamato et al. (2.36 ± 0.72 mg/lung). Lee et al. (1981) exposed rats, hamsters and guinea pigs to up to 101,500 fibers/cm^3^ for 6 h/d, 5 d/wk. for 3 months: lung burdens were not determined [5]. In our TIPS instillation study, we administered 0.25 and 0.50 mg POT fibers to male F344 rats, which resulted in an initial lung exposure of approximately 750 and 1500 fibers/μg dry weight. The exposure level in our study is similar to the minimum level of exposure to crocidolite asbestos that resulted in tumor development in the rat lung, 1250 fibers/μg dry weight [20], and the induction of lung tumors is also similar to that caused by this level of asbestos exposure, 15 lung tumors in 106 rats in the asbestos study and 1 lung tumor in 17 rats exposed to 0.25 mg POT fibers and 2 lung tumors in 16 rats exposed to 0.50 mg POT fibers. Therefore, the most probable explanation for the different results of our study from those of Ikegami, Yamato, and Oyabu is that POT fibers did not accumulate to high enough levels for a sufficiently long period of time to induce tumorigenesis. The reason for the negative result in the Lee study is not known, but nasal filtering may have been a factor. The nasal passages of rats are an efficient particle filter and impact particle deposition [21]. Nasal filtering would have also impacted particle deposition in the studies by Ikegami, Yamato, and Oyabu. Finally, it is notable that in the study by Yamato et al. (2003), 2.2 mg of POT fibers per m^3^ was equivalent to 111 POT fibers per cm^3^ [10]. Thus, 1 mg of the material used in the Yamato et al. (2003) study contained approximately 50 × 10^6^ fibers. In contrast, in our study, 1 mg of material contained approximately 646 × 10^6^ fibers (see Additional file 5). Thus, either the POT fibers were larger or more aggregated in the Yamato et al. (2003) study than in our study.

In this study, MWCNT-7, a known lung carcinogen in rats, was used as a positive control reference material. MWCNT-7 was administered at a level well below that which accumulated in the lungs of male rats exposed by inhalation to 2 mg/m^3^ MWCNT-7 for 2 years (1.8 mg/lung) [16]. The incidence of lung carcinomas that developed in male rats exposed to 2 mg/m^3^ MWCNT-7 in the inhalation study was 11/50, and the incidence was 5/14 in the rats administered 0.50 mg MWCNT-7 in the present study. The incidence of pleural mesotheliomas was 0/50 in the inhalation study and 2/14 in the present study. Thus, while the incidences of malignant tumor development in the lung and pleura in the MWCNT-7 group in the present study were not statistically higher than in the control groups, malignant tumor development in the MWCNT-7 treated rats was similar to malignant tumor development in rats exposed to 2 mg/m^3^ MWCNT-7, and as discussed above, the malignant tumors that developed in the MWCNT-7 group were highly likely to be treatment related.

Both POT and MWCNT-7 fibers, but not r-nTiO_2_, were biopersistent in the lung and pleura and were observed interacting with macrophages at 3 weeks, 1 year, and 2 years after TIPS administration of the test materials into the lung (see Abdelgied 2019 [12] and Table 2 and Fig. 2). These attributes are central to fiber-carcinogenicity. We propose the following mechanism to explain the carcinogenicity of POT and MWCNT-7 fibers. Biopersistence of POT and MWCNT-7 fibers resulted in persistent generation of reactive oxygen and nitrogen species (ROS and RNS) and other inflammatory mediators by macrophages attempting to clear these fibers out of the lung and pleural cavity. The tissue damage caused by this inflammatory response and the damage caused by the fibers themselves resulted in a tissue repair response: as shown in Abdelgied et al. 2018 [11] and Abdelgied et al., 2019 [12] the PCNA index at 4 weeks and at 1 year in the lung and pleura of rats treated with POT or MWCNT-7 fibers, but not r-nTiO_2_, was significantly higher than that of the control groups. Persistence of the fibers at the site of tissue injury would result in the generation of DNA-damaging oxidants in the presence of cells replicating in response to tissue damage. Thus, the DNA damaging ROS and RNS could have damaged the DNA of dividing cells, allowing replication of damaged DNA before it was repaired, resulting in fixation of mutations in the DNA of the daughter cells. Eventually, repeated cycles of tissue damage and tissue repair in the presence of DNA damaging agents could lead to the accumulation of mutations that would allow a cell to bypass tissue and cellular checkpoints and initiate the neoplastic transformation process. (Please see Additional file 6 for a discussion of how this mechanism is proposed to operate in the context of asbestos-induction of malignant mesothelioma.)

## Conclusions

Four of 33 rats administered POT fibers via TIPS developed pleural mesothelioma, which is significantly higher than the JBRC historical control data (3 of 3398 rats spontaneously developed malignant mesothelioma). Thus, based on JBRC historical control data, POT fibers are carcinogenic to the pleura of male rats when administered to the lung via the airway using TIPS. The incidence of type II epithelial cell hyperplasia and the histology of the carcinomas that developed in the POT treated rats also indicates that POT fibers are highly likely to be carcinogenic in the lung of male rats. These results are in agreement with the physical characteristics of these thin, long, biopersistent fibers. Intratracheal instillation studies are used for hazard identification and ranking of hazardous respirable materials [22–24]. The results of the present study suggest that POT fibers are a potential hazard to human health.

## Methods

### Preparation of the test materials

r-TiO_2_, POT fibers, and MWCNT-7 (Mitsui Chemicals Inc., Tokyo, Japan) were supplied by Dr. A. Hirose (one of the authors). The three test materials were dispersed by the Taquann method [19]. The dispersed material was stored in tert-butyl alcohol. Shortly before administration, the frozen T-butyl alcohol was removed using an Eyela Freeze Drying machine (FDU-2110; Tokyo Rikakikai Co., Ltd., Tokyo, Japan). The test materials were then dispersed in saline containing 0.5% poloxamer-188 (P5556: Sigma-Aldrich, St. Louis, MO, USA): P5556, a 10% solution of poloxamer 188, was diluted 1:20 in physiological saline. Poloxamer 188 was used to keep the test materials used in this study in suspension. The test materials were dispersed at concentrations of 62.5 and 125 μg/ml (POT and r-nTiO2) and 125 μg/ml (MWCNT-7) and then sonicated for 30 min using an SFX250 bench top sonicator at 250 watts and a frequency of 20 kHz. (Sonifier®, Emerson Electric Asia-Pacific, Hong Kong). To minimize aggregation, after transfer to the animal rooms the suspensions were kept in a Bransonic M1800-J bath sonicator (Branson Ultrasonics Co., Ltd., Shanghai, China).

### Characterization of the test materials

After dispersion of the test material in saline/poloxamer-188 and subsequent sonication, 20 μl of the suspension was placed on a micro grid membrane pasting copper mesh (EMS 200-Cu, Nisshin EM Co., Ltd., Tokyo, Japan). The shape of the nanoparticles was imaged by transmission electron microscopy (JEOL Co. Ltd., Tokyo, Japan), and the photos were analyzed by NIH image analyzer software (NIH, Bethesda, Maryland, USA). Over 1000 particles of each type of material were measured. The test materials were also examined after being aerosolized, i.e., sprayed out of the microsprayer used for TIPS administration of the material: TIPS administration using the microsprayer is described below. Element analysis was done using a Scanning electron microscope with an X-ray microanalyzer (ADEX, Tokyo, Japan).

### Animals

Nine-week old male F344/DuCrjCrlj rats were purchased from Charles River Japan Inc. (Yokohama, Japan). The rats were housed in the animal center of Nagoya City University Medical School, maintained on a 12 h light-dark cycle, and received oriental MF basal diet (Oriental Yeast Co., Tokyo, Japan) and water ad libitum. The experimental protocol was approved by the Animal Care and Use Committee of Nagoya City University Medical School, and the research was conducted according to the Guidelines for the Care and Use of Laboratory Animals of Nagoya City University.

### Intra-tracheal intra-pulmonary spraying (TIPS)

A 1 ml syringe attached to a Penn-Century Intratracheal Aerosolizer (model 1A-1B-C) was used for TIPS administration of the vehicle and test materials. (Penn-Century, Inc. is closed and no longer sells these microsprayers. Microsprayers are available from DIMS Institute of Medical Science, Inc., 64 Goura, Nishiazai, Azai-cho, Ichinomiya 491–0113, Japan). Rats were placed under 3% isoflurane anesthesia, and the anesthetized rat was maintained in a vertical position. During inspiration the trachea opens and the nozzle of the microsprayer was inserted into the open trachea and 0.5 ml of the vehicle/test material was sprayed into the lungs. The microsprayer was then removed and the rat was maintained in a vertical position until it recovered from the instillation procedure.

### Experimental design

After acclimatization for 2 weeks, 210 rats were divided into 7 groups of 30 animals each: group 1, without treatment; group 2, administered vehicle (saline plus 0.5% poloxamer-188 solution); group 3, administered 0.25 mg r-nTiO_2_; group 4, administered 0.50 mg r-nTiO_2_; Group 5, administered 0.25 mg POT fibers; Group 6, administered 0.50 mg POT fibers; and Group 7, administered 0.50 mg MWCNT-7. Rats were administered 0.5 ml vehicle, 62.5 μg/ml and 125 μg/ml r-nTiO2 and POT fibers, and 125 μg/ml MWCNT-7 once every other day over a 15-day period (8 doses). All animals that died before the end of the experiment were necropsied immediately on discovery. Three weeks and 52 weeks after the start of the experiment interim sacrifices were performed: 5 rats from each group were killed by exsanguination from the abdominal aorta under deep isoflurane anesthesia [11, 12]. Animals found moribund after the 52 week sacrifice and animals that survived to the end of the experimental period, 105 weeks from the start of experiment, underwent terminal necropsy: terminal sacrifice was performed by exsanguination from the abdominal aorta under deep isoflurane anesthesia.

### Tissue sample collection, organ weights, pleural cavity lavage collection, and pathological examination

Animals were observed daily for clinical signs and mortality. Body weights were measured weekly throughout the experimental period. At necropsy, blood samples were collected via the abdominal aorta under deep isoflurane anesthesia and serum samples were stored at − 80 °C. Organs, including lung, liver, kidney, spleen, brain, heart, and testes were weighed and examined for any macroscopic lesions. The trachea, esophagus, lymph nodes (including mediastinal lymph nodes), epididymis, seminal vesicles, prostrate, seminal vesicle, urinary bladder, intestines, spinal bone, thoracic wall, eyes, skin lesions, nasal cavity, pancreas, muscles, and diaphragm (which includes the diaphragmatic region of the parietal pleura) were examined macroscopically then processed and examined histopathologically. Tissue sections were evaluated by two board-certified pathologists, Hiroyuki Tsuda and Satoru Takahashi, two of the authors. Collagen deposition was confirmed using polarized light microscopy. Five rats from each group were randomly selected for pleural cavity lavage collection. No macroscopic lung tumors were observed in any of these rats, and the lungs were excised and the right upper and middle lobes were cut into pieces and processed for measuring the length and diameter of test materials by SEM (see below). The remaining right lobes and the left lung as well as the lungs of the remaining animals were inflated and fixed with 4% paraformaldehyde solution in phosphate-buffered saline (PBS) adjusted to pH 7.3 and processed for light microscopic examination. Both the nasal cavity and thoracic wall were decalcified in formic acid-formalin solution and then trimmed and processed for light microscopic examination.

### Observation of test materials in the lung and mediastinal LNs

Both lung tissue (1 cm^3^ in volume) and mediastinal LNs were digested according to the method of Kohyama and Suzuki. Briefly, tissues were allowed to react with the digestion solution, Clean 99-K200® (Clean chemical Co., Ltd., Ibraki, Japan), overnight. Then the digested solution was centrifuged at 12,000 rpm for 30 min and the supernatant was discarded. The pellet was resuspended in distilled water and lightly sonicated. The pellet was collected by centrifugation and washed two more times. After a final centrifugation, the pellet was resuspended in 200 μl of distilled water and the specimens were collected on EMD Millipore™ Polycarbonate Membrane Filters (Millipore, Tokyo, Japan) and observed by SEM (Field Emission Scanning Electronic Microscope; Hitachi High Technologies, Tokyo, Japan) at 5–10 kV.

### Measurement of the morphometry of test materials and biochemical examination of pleural cavity lavage fluid (PLF)

At the terminal necropsy, PLF was collected from 5 rats randomly selected from each group; PLF was collected as previously described [11].

One ml of the PLF was used to collect test materials and measure the morphometry. The PLF was briefly sonicated then centrifuged at 3000 rpm for 10 min. The supernatant was removed and the pellet was digested according to Kohyama and Suzui [25], collected on EMD Millipore™ Polycarbonate Membrane Filters, and observed by SEM as described above. The number and dimensions of particles/fibers were measured.

Lactate dehydrogenase (LDH) activity was measured in the PLF using an LDH activity assay kit (Sigma-Aldrich, St Louis, MO, USA) and total protein concentration was measured using the BCA Protein assay kit (Pierce biotech, Rockford, IL, USA).

### Statistical analysis

The incidences of neoplastic, preneoplastic, and non-neoplastic lesions in the lung and pleura were analyzed for significant difference from vehicle treated control by Fisher’s exact test. Body weight, organ weights, and the results of the biochemical analysis of the PLF were analyzed for homogeneity of variance using Levene’s test. The variance was homogeneous, therefore, the data was analyzed by Dunnett’s multiple comparison test. Survival curves were plotted according to the method of Kaplan-Meier [26], and the log-rank test was used to detect statistically significant differences between test material treated groups and the vehicle treated control group. SPSS software version 25 (IBM, Armonk, New York, USA) was used. *P* values < 0.05 were considered to be statistically significant.

## Additional files

Additional file 1: A Brief Discussion of In Vivo POT Fiber Studies. (PDF 265 kb)

Additional file 2: Characterization of test materials used in this study. **Figure S1.** TEM images of r-nTiO_2_, POT fibers, and MWCNT-7. **Figure S2.** Lengths and diameters of the POT fibers used in this study. (PDF 3149 kb)

Additional file 3: **Table S1.** Disposition of the animals used in this study. **Table S2.** Non-treatment-related deaths prior to week 73. **Table S3.** Final body weights. **Table S4.** Neoplastic lesions found in individual rats. **Table S5.** Incidence of other tumors. **Table S6.** Absolute organ weights. **Table S7.** Relative organ weights. **Table S8.** Non-neoplastic lesions in the lung and pleura. **Table S9.** LDH and Total Protein found in the pleural lavage fluid of individual rats. (PDF 531 kb)

Additional file 4: Survival curves, body weights, and fibers in the mediastinal lymph nodes of rats administered POT and MWCNT-7 fibers. **Figure S1.** Kaplan Meier survival curves. **Figure S2.** Body weight curves. **Figure S3.** Mediastinal lymph nodes of rats treated with POT and MWCNT-7 fibers. (PDF 5648 kb)

Additional file 5: Lung Fiber Burdens in 2-year POT Studies. (PDF 176 kb)

Additional file 6: Lessons from Malignant Pleural Mesothelioma. (PDF 319 kb)

## Data Availability

All data generated or analysed during this study are included in this published article [and its additional files].

## Abbreviations

a-nTiO_2_: Anatase titanium dioxide nanoparticles
LDH: Lactate dehydrogenase
MWCNT: Multi-walled carbon nanotube
PLF: Pleural lavage fluid
POT: Potassium octatitanate
r-nTiO_2_: Rutile titanium dioxide nanoparticles
SEM: Scanning electron microscopy
TEM: Transmission electron microscopy
TiO_2_: Titanium dioxide
TIPS: Intra-tracheal intra-pulmonary spraying
TP: Total protein
