# Supplementary material for: Carcinogenic effect of potassium octatitanate (POT) fibers in the lung and pleura of male Fischer 344 rats after intrapulmonary administration
Source: Part Fibre Toxicol. 2019 Sep 2;16:34. doi: 10.1186/s12989-019-0316-2 (PMC6720102; doi:10.1186/s12989-019-0316-2)
Supplement: Supplementary file 1 — A Brief Discussion of In Vivo POT Fiber Studies. (PDF 265 kb) [file 12989_2019_316_MOESM1_ESM.pdf]

A Brief Discussion of *In Vivo* POT Fiber Studies

The fiber pathogenicity paradigm identifies three critical factors that dictate whether or not an inhaled fiber is potentially pathogenic [1]. These factors are width, length, and biopersistence. Width affects fiber rigidity and aerodynamic diameter, and therefore, width affects the deposition pattern of the fiber. Length affects the ability of macrophages to physically remove fibers that are deposited beyond the ciliated airways. Biopersistence is a measure of the physical stability of deposited fibers. POT fibers have a long needle-like shape, similar to asbestos, and accordingly, they have aerodynamic diameters that allow deposition beyond the ciliated airways and lengths that inhibit clearance by macrophages. POT fibers are also biopersistent: POT fibers administered to rats by inhalation can be recovered 12 months after administration without any erosion on their surface [2]. Thus, the fiber pathogenicity paradigm identifies these fibers as potential carcinogens.

The carcinogenicity of POT fibers was confirmed by an initial study that administered POT fibers by application of hardened gelatin containing 40 mg POT fibers directly to the pleural surface of Osborne-Mendel rats [3, 4]. This study followed the treated animals for up to 2 years and found that approximately 70% of the rats developed pleural sarcomas (Table 1). A later 2-year study using intraperitoneal injection of rats with POT fibers found that injection of 5 mg and 10 mg POT fibers resulted in cumulative incidences of peritoneal mesothelioma of 20% and 77%, respectively (Table 1) [5]. Based in part on the biopersistence of POT fibers and the fact that intraperitoneal injection of POT fibers resulted in the development of mesothelioma in rats, the WHO Workshop on Mechanisms of Fibre Carcinogenesis and Assessment of Chrysotile Asbestos Substitutes concluded that respirable potassium octatitanate fibers are likely to pose a high hazard to humans after inhalation exposure [6].

Four additional more recently conducted studies infused POT fibers directly into the thoracic cavity of mice and rats (Table 1) [7, 8, 9, 10]. These studies followed the treated animals for 21 weeks and for approximately 1 year. The studies that terminated at 21 weeks reported pleural thickening, and the studies that terminated at approximately 1 year reported chronic inflammation and proliferation of pleural mesothelial cells. Mesotheliomas did not develop in any of the treated animals. These studies suggest that the carcinogenic effect of POT fibers may require extended exposure of susceptible tissues to the fibers.

Table 1

Administration	Dose	Test Animal	Study Period	Treatment Related Tumors	Ref
Intrapleural implantation	40 mg<br><br>40 mg	Rats<br><br>Rats	2 yr<br><br>2 yr	21/29 pleural sarcomas<br><br>20/29 pleural sarcomas	3,4*
Intraperitoneal injection	5 mg<br><br>10 mg	Rats<br><br>Rats	2 yr<br><br>2 yr	20% mesothelioma<br><br>77% mesothelioma	5
Infusion into the left pleural cavity	1.5 mg	Mice	21 wk	No tumors reported	7
Infusion into the left pleural cavity	3 mg	Mice	21 wk	No tumors reported	8
Infusion into the left pleural cavity	3 mg	Mice	52 & 65 wk	No	9
Infusion into the left pleural cavity	3 mg<br><br>30 mg<br><br>30 mg + DHPN	Mice<br><br>Rats<br><br>Rats	415 days<br><br>1 yr<br><br>1 yr	No<br><br>No<br><br>No increase over DHPN alone	10

* Ref 3 and 4 both indicate a 100% chance of developing tumors. Ref 4 gives the actual tumor incidences, which are shown in the table.

Notably, the studies cited above administered extremely high doses of fibers into the pleural and peritoneal cavities. These levels would accumulate in the pleural cavities of test animals only after respiratory exposure to exceedingly high levels of fibers. For example, in rats exposed for 2 years to 2 mg/m^3 MWCNT-7, the total fiber burden in the pleura was approximately 1468 and 847 fibers in males and females while the total fiber burden in the lung was 16.2×10^9 and 10.4×10^9 fibers in males and females [11]. While the ratio of lung to

pleural fiber burden is not expected to be the same for MWCNT-7 and POT fibers, these results do support the premise that exposure via the airway results in substantially greater fiber burdens in the lung than in the pleural cavity. Overall, it is unlikely that the levels of fibers used in the studies cited above could accumulate in the pleural cavities of test animals exposed to POT fibers via inhalation.

Several studies exposed test animals to POT fibers via the airway and followed the animals for at least 6 months. However, most of these studies were terminated between 6 months and approximately 1 year (Table 2) [2, 12, 13, 14, 15, 16, 17, 18].

Table 2

Administration	Dose	Test Animal	Study Period	Treatment Related Tumors	Ref
Whole Body Inhalation: 6 h/day, 5 d/wk, 13 wk (Ikegami)	1 mg/m^3 (1700 $fibers/cm^3$, 123 WHO $fibers/cm^3$)	Rats	6 mo	No tumors reported	12
	10 mg/m^3 (5900 $fibers/cm^3$, 952 WHO $fibers/cm^3$)	Rats	6 mo	No tumors reported	
	100 mg/m^3 (112,700 $fibers/cm^3$, 7440 WHO $fibers/cm^3$)	Rats	6 mo	No tumors reported	
Whole Body Inhalation: 6 h/day, 5 d/wk, 1 yr (Morimoto)	2.2 mg/m^3 (111 $fibers/cm^3$)	Rats	1 yr	No tumors reported	13
Whole Body Inhalation: 6 h/day, 5 d/wk, 4 wk (Yamato)	1.1 mg/m^3 and 1.7 mg/m^3 (137 & 73 $fibers/m^3$ [sic])	Rats	13 mo	No tumors reported	2
Intratracheal Instillation (single) Ding	2, 10 mg	Rats	6 mo	No tumors reported	14
Intratracheal Instillation (single) Oyabu	0.5, 1, 2, 5 mg	Rats	1 yr	No tumors reported	15
Intratracheal Instillation (single) Morimoto	2 mg	Rats	6 mo	No tumors reported	16
Intratracheal Instillation (single) Ogami	2 mg	Rats	6 mo	No tumors reported	17
Intratracheal Instillation (single) Obata	1, 2 mg	Rats	6 mo	No tumors reported	18

In agreement with the pleural cavity infusion studies, none of the studies that followed the exposed animals for less than 2 years reported the development of lung or pleural tumors.

Four studies exposed animals to POT fibers by inhalation and followed the exposed animals for up to 2 years (Table 3). Lee et al. (1981) exposed rats, hamsters, and guinea pigs to up to 371 mg/m^3 POT fibers for 6 h/day, 5 days/wk for 3 months and followed the animals for an additional 15 - 24 months (fibers longer than 5 μM and less than 10 μM were approximately 35-45% of the total fibers) [19]. Yamato et al. (2003) exposed rats to 2.2 mg/m^3 (111 $fibers/cm^3$) POT fibers for 6 h/day, 5 days/wk for 1 year and followed the animals for up to 1 year after the end of the exposure period [20]. Oyabu et al. (2004) exposed rats to 1.9 mg/m^3 POT fibers for 6 h/day, 5 days/wk for 1 year and followed the animals for up to 1 year after the end of the exposure period [21]. The calculated amount of POT fibers inhaled in the Yamato et al. (2003) and Oyabu et al. (2004) studies was approximately 30 mg [21]. Ikegami et al. (2004) exposed rats to up to 200 POT $fibers/cm^3$ for 6 h/day, 5 days/wk for 24 months [22].

Yamato et al. (2003), Oyabu et al. (2004), and Ikegami et al. (2004) report that POT fiber exposed rats did not develop exposure related malignant neoplasms. Yamato et al. (2003) did report the development of non-malignant neoplasms in POT fiber exposed rats: 2 of 5 rats had developed lung adenomas 6 months after the end of the one year exposure period and 1 of 11 rats developed a lung adenoma and 1 rat developed squamous metaplasia at 12 months after the end of the exposure period, however, this incidence was not statistically significant. Rats exposed to POT fibers in the studies reported by Oyabu et al. (2004) and Ikegami et al. (2004), studies using very similar conditions to those of Yamato et al. (2003), did not develop any exposure related proliferative lesions. Only Lee et al. (1981), a study that used much higher levels of POT fibers than the other inhalation studies, reported the development of a malignant neoplasm in POT fiber exposed animals. In this study, one hamster in the group exposed to 79 mg/m^3, one hamster in the group exposed to 82 mg/m^3, and one hamster in the group exposed to 371 mg/m^3 developed mesothelioma. No mesotheliomas were observed in POT fiber exposed rats or guinea pigs. A few other pulmonary tumors developed in the exposed animals, but the incidence was not higher than in the control groups. Notably, the development of pleural tumors but not pulmonary tumors in hamsters in the study by Lee et al. (1981) may have been specific to hamsters [23, 24].

Table 3. Summary of Two-Year Inhalation Studies

Fiber Concentration (mg/m³)	Fiber Number (fibers/cm³)	Exposure	Malignant Tumor Development	
79<br>39<br>82<br>371	8,530*<br>6,720*<br>36,100*<br>101,500*	6 h/day, 5 d/wk, 3 months	Hamsters - Yes<br>Guinea pigs - No<br>Rats - No	Lee 1981
2.2	111	6 h/day, 5 d/wk, 1 yr	No	Yamato 2003
1.9	Not Stated	6 h/day, 5 d/wk, 1 yr	No	Oyabu 2004
Not Stated	Up to 200	6 h/day, 5 d/wk, 2 yr	No	Ikegami 2004

* Total fiber numbers

Overall, the studies cited here indicate that POT fibers are carcinogenic, but that a high amount of POT fibers in prolonged contact with susceptible tissue is required for induction of malignant neoplasia. Importantly, as can be seen in Table 3, the 2-year inhalation studies using a maximum of 100 - 200 fibers/cm^3 did not give rise to malignant lung or pleural tumors. Even the study by Lee et al. (1981), which exposed test animals to up to 101,500 fibers/cm^3, did not find any exposure-related neoplasms in rats or guinea pigs. These studies suggest that inhalation of POT fibers is not carcinogenic in rats and that low level exposure may not be carcinogenic in humans [22].

References

1. Donaldson K, Murphy F, Schinwald A, Duffin R, Poland CA. Identifying the pulmonary hazard of high aspect ratio nanoparticles to enable their safety-by-design. Nanomedicine (Lond). 2011;6 1:143-56; doi: 10.2217/nnm.10.139. http://www.ncbi.nlm.nih.gov/pubmed/21182425.
2. Yamato H, Morimoto Y, Tsuda T, Ogami A, Oyabu T, Ishimatsu S, et al. Clearance of Inhaled Potassium Octatitanate Whisker from Rat Lungs. Journal of occupational health. 2002;44 1:34-9.
3. Stanton MF, Layard M. The carcinogenicity of fibrous minerals. In: Workshop on Asbestos: Definitions and Measurement Methods. National Bureau of Standards Special Publication 506. 1978: 143-51. https://nvlpubs.nist.gov/nistpubs/Legacy/SP/nbsspecialpublication506.pdf#page=153 Accessed 04 June 2019.
4. Stanton MF, Layard M, Tegeris A, Miller E, May M, Morgan E, et al. Relation of particle dimension to carcinogenicity in amphibole asbestoses and other fibrous minerals. J Natl Cancer Inst. 1981;67 5:965-75. http://www.ncbi.nlm.nih.gov/pubmed/6946253.
5. Adachi S, Kawamura K, Takemoto K. A trial on the quantitative risk assessment of man-made mineral fibers by the rat intraperitoneal administration assay using the JFM standard fibrous samples. Ind Health. 2001;39 2:168-74. http://www.ncbi.nlm.nih.gov/pubmed/11341547.
6. WHO. Workshop on Mechanisms of Fibre Carcinogenesis and Assessment of Chrysotile Asbestos Substitutes 8-12 November 2005, Lyon, France. Summary Consensus Report. https://www.who.int/ipcs/publications/new_issues/summary_report.pdf Accessed 22 Oct 2018.
7. Yokohira M, Hashimoto N, Yamakawa K, Suzuki S, Saoo K, Kuno T, et al. Potassium octatitanate fibers (TISMO) induce pleural mesothelial cell reactions with iron accumulation in female A/J mice. Oncol Lett. 2010;1 4:589-94; doi: 10.3892/ol_00000104. https://www.ncbi.nlm.nih.gov/pubmed/22966348.
8. Yokohira M, Nakano Y, Yamakawa K, Kishi S, Ninomiya F, Saoo K, et al. Strain differences in pleural mesothelial cell reactions induced by potassium octatitanate fibers (TISMO) infused directly into the thoracic cavity. Exp Toxicol Pathol. 2013;65 6:925-32; doi: 10.1016/j.etp.2013.01.006. https://www.ncbi.nlm.nih.gov/pubmed/23375775.
9. Yokohira M, Hashimoto N, Nakagawa T, Nakano Y, Yamakawa K, Kishi S, et al. Long-Term Chronic Toxicity and Mesothelial Cell Reactions Induced by Potassium Octatitanate Fibers (TISMO) in the Left Thoracic Cavity in A/J Female Mice. Int J Toxicol. 2015;34 4:325-35; doi: 10.1177/1091581815587744. https://www.ncbi.nlm.nih.gov/pubmed/26023052.
10. Yokohira M, Nakano-Narusawa Y, Yamakawa K, Hashimoto N, Yoshida S, Kanie S, et al. Chronic mesothelial reaction and toxicity of potassium octatitanate fibers in the pleural cavity in mice and F344 rats. Cancer Sci. 2016;107 7:1047-54; doi: 10.1111/cas.12944. http://www.ncbi.nlm.nih.gov/pubmed/27088262.

11. Kasai T, Umeda Y, Ohnishi M, Mine T, Kondo H, Takeuchi T, et al. Lung carcinogenicity of inhaled multi-walled carbon nanotube in rats. Part Fibre Toxicol. 2016;13 1:53; doi: 10.1186/s12989-016-0164-2. http://www.ncbi.nlm.nih.gov/pubmed/27737701.
12. Ikegami T, Taniguchi M, Singer AW, Brooker MJ, Yarrington J, Placke ME, et al. Inhalation toxicity of potassium octatitanate fibers (TISMO) in rats following 13 weeks of aerosol exposure. Inhal Toxicol. 2000;12 5:415-38; doi: 10.1080/089583700196121. https://www.ncbi.nlm.nih.gov/pubmed/10880137.
13. Morimoto Y, Tsuda T, Yamato H, Oyabu T, Higashi T, Tanaka I, et al. Comparison of gene expression of cytokines mRNA in lungs of rats induced by intratracheal instillation and inhalation of mineral fibers. Inhal Toxicol. 2001;13 7:589-601; doi: 10.1080/08958370120008. https://www.ncbi.nlm.nih.gov/pubmed/11452356.
14. Ding L, Morimoto Y, Oyabu T, KIM H, OHGAMI A, YATERA K, et al. Gene expression of Clara cell secretory protein, surfactant protein-A and thyroid transcription factor-1 in the lungs of rats exposed to potassium octatitanate whiskers in vivo. Journal of Occupational Health. 2001;43 3:111-7.
15. Oyabu T, Yamato H, Ogami A, Morimoto Y, Akiyama I, Ishimatsu S, et al. The effect of lung burden on biopersistence and pulmonary effects in rats exposed to potassium octatitanate whiskers by intratracheal instillation. J Occup Health. 2006;48 1:44-8; doi: 10.1539/joh.48.44. https://www.ncbi.nlm.nih.gov/pubmed/16484762.
16. Morimoto Y, Ogami A, Nagatomo H, Hirohashi M, Oyabu T, Kuroda K, et al. Calcitonin gene-related peptide (CGRP) as hazard marker for lung injury induced by dusts. Inhal Toxicol. 2007;19 3:283-9; doi: 10.1080/08958370601069364. https://www.ncbi.nlm.nih.gov/pubmed/17365031.
17. Ogami A, Morimoto Y, Myojo T, Oyabu T, Murakami M, Nishi K, et al. Histopathological changes in rat lung following intratracheal instillation of silicon carbide whiskers and potassium octatitanate whiskers. Inhal Toxicol. 2007;19 9:753-8; doi: 10.1080/08958370701399869. http://www.ncbi.nlm.nih.gov/pubmed/17613083.
18. Obata Y, Morimoto Y, Hirohashi M, Ogami A, Oyabu T, Myojo T, et al. Expression of heme oxygenase-1 in the lungs of rats exposed to potassium octatitanate whiskers. J Occup Health. 2011;53 4:267-73; doi: 10.1539/joh.l10056. https://www.ncbi.nlm.nih.gov/pubmed/21670561.
19. Lee KP, Barras CE, Griffith FD, Waritz RS. Pulmonary response and transmigration of inorganic fibers by inhalation exposure. Am J Pathol. 1981;102 3:314-23. https://www.ncbi.nlm.nih.gov/pubmed/7212016.
20. Yamato H, Oyabu T, Ogami A, Morimoto Y, Higashi T, Tanaka I, et al. Pulmonary effects and clearance after long-term inhalation of potassium octatitanate whiskers in rats. Inhal Toxicol. 2003;15 14:1421-34; doi: 10.1080/08958370390248969. http://www.ncbi.nlm.nih.gov/pubmed/14648357.
21. Oyabu T, Yamato H, Ogami A, Morimoto Y, Akiyama I, Ishimatsu S, et al. The effect of lung burden on biopersistence and pulmonary effects in rats exposed to potassium octatitanate whiskers by inhalation. J Occup Health. 2004;46 5:382-90. http://www.ncbi.nlm.nih.gov/pubmed/15492455.

22. Ikegami T, Tanaka A, Taniguchi M, Clark M, Ragan H, Mast T, et al. Chronic inhalation toxicity and carcinogenicity study on potassium octatitanate fibers (TISMO) in rats. Inhal Toxicol. 2004;16 5:291-310; doi: 10.1080/08958370490428391. http://www.ncbi.nlm.nih.gov/pubmed/15371181.
23. Bermudez E, Mangum JB, Wong BA, Asgharian B, Hext PM, Warheit DB, et al. Pulmonary responses of mice, rats, and hamsters to subchronic inhalation of ultrafine titanium dioxide particles. Toxicol Sci. 2004;77 2:347-57; doi: 10.1093/toxsci/kfh019. https://www.ncbi.nlm.nih.gov/pubmed/14600271.
24. Gelzleichter TR, Bermudez E, Mangum JB, Wong BA, Janszen DB, Moss OR, et al. Comparison of pulmonary and pleural responses of rats and hamsters to inhaled refractory ceramic fibers. Toxicol Sci. 1999;49 1:93-101. https://www.ncbi.nlm.nih.gov/pubmed/10367346.
