# Supplementary material for: Carcinogenic effect of potassium octatitanate (POT) fibers in the lung and pleura of male Fischer 344 rats after intrapulmonary administration
Source: Part Fibre Toxicol. 2019 Sep 2;16:34. doi: 10.1186/s12989-019-0316-2 (PMC6720102; doi:10.1186/s12989-019-0316-2)
Supplement: Supplementary file 2 — Characterization of test materials used in this study. Figure S1. TEM images of r-nTiO2, POT fibers, and MWCNT-7. Figure S2. Lengths and diameters of the POT fibers used in this study. (PDF 3149 kb) [file 12989_2019_316_MOESM2_ESM.pdf]

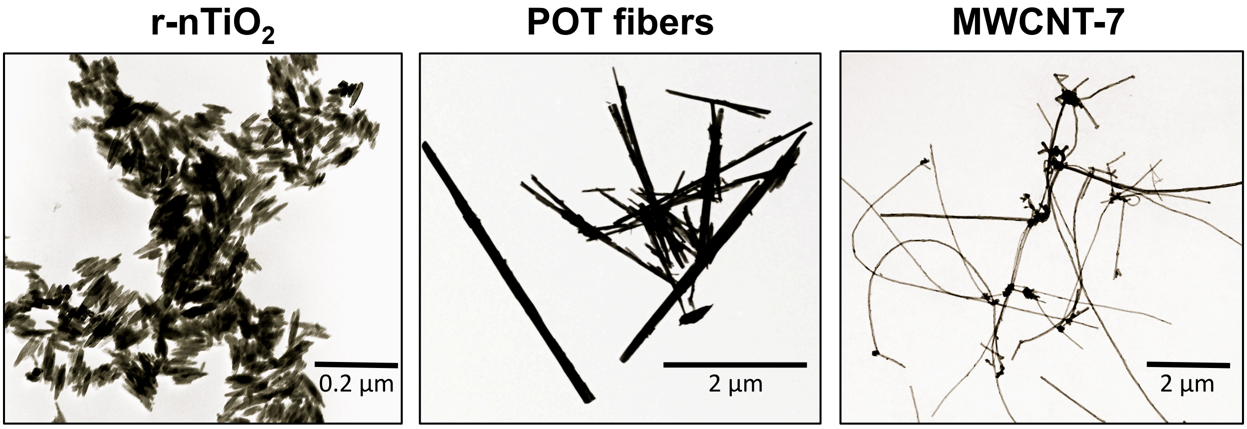

Figure S1 TEM images of r-nTiO_2, POT fibers, and MWCNT-7.

Figure S1 is based on Supplementary Figure S2 in Supplementary Doc S2 in Abdelgied et al, 2019 [11]: Creative Commons Attribution 4.0 International License (http://creativecommons.org/licenses/by/4.0/).

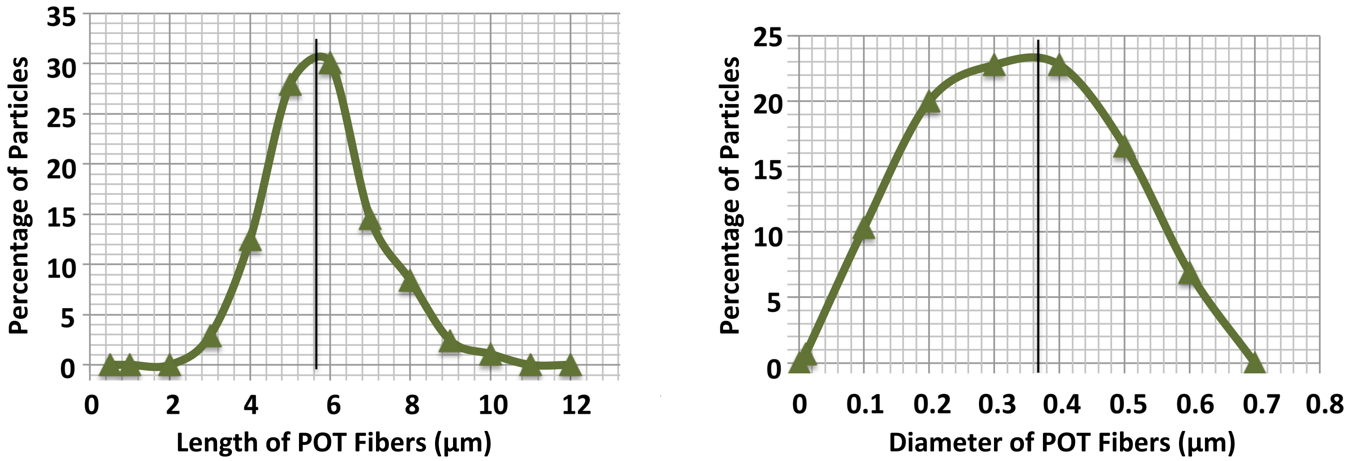

Figure S2 Lengths and diameters of the POT fibers used in this study.

Figure S2 is dreived from Supplementary Figure S1 in Abdelgied et al, 2018 [12]: Creative Commons Attribution 4.0 International License (http://creativecommons.org/licenses/by/4.0/).
