# Supplementary material for: Carcinogenic effect of potassium octatitanate (POT) fibers in the lung and pleura of male Fischer 344 rats after intrapulmonary administration
Source: Part Fibre Toxicol. 2019 Sep 2;16:34. doi: 10.1186/s12989-019-0316-2 (PMC6720102; doi:10.1186/s12989-019-0316-2)
Supplement: Supplementary file 3 — Table S1. Disposition of the animals used in this study. Table S2. Non-treatment-related deaths prior to week 73. Table S3. Final body weights. Table S4. Neoplastic lesions found in individual rats. Table S5. Incidence of other tumors. Table S6. Absolute organ weights. Table S7. Relative organ weights. Table S8. Non-neoplastic lesions in the lung and pleura. Table S9. LDH and Total Protein found in the pleural lavage fluid of individual rats. (PDF 531 kb) [file 12989_2019_316_MOESM3_ESM.pdf]

Table S1: Disposition of the animals used in this study.

Group	Starting animal number	Animals entered into the study[a]	Animals sacrificed at 3 weeks	Animals sacrificed at 1 year	Initial number of animals for the 2-year end-point	Survival to week 73[b]	Survival to week 105	Mean survival time (weeks)
Untreated	30	30	5	5	20	19	15	101.80 ± 2.166
Vehicle	30	30	5	5	20	19	13	100.95 ± 1.858
r-nTiO_2 (0.25 mg)	30	30	5	5	20	20	14	102.60 ± 1.003
r-nTiO2 (0.50 mg)	30	29	5	5	19	19	17	104.16 ± 0.637
POT (0.25 mg)	30	28	5	5	18	17	9	99.89 ± 2.289
POT (0.50 mg)	30	29	5	5	19	16	11	92.89 ± 4.098
MWCNT-7 (0.50 mg)	30	30	5	5	20	14	8	87.73 ± 4.635*

[a] Four rats died during TIPS administration: 1 rat in the 0.50 mg r-nTiO_2 group, 2 rats in the 0.25 POT group, and 1 rat in the 0.50 mg POT group.
[b] Prior to the first treatment-related death at week 73, an additional 12 rats died from non-treatment related causes. These 12 rats were excluded from the study.
* Different from the Vehicle Control at p<0.05.

Table S2: Non-treatment-related deaths prior to week 73.

Group	Animal number	Cause of Death
Untreated	1	Leukemia (day 426)
Vehicle	1	Food clogging (day 478)
r-nTiO_2 (0.25 mg)	0	
r-nTiO2 (0.50 mg)	1	Died during TIPS administration of the test material.
POT (0.25 mg)	3	2 rats died during TIPS administration of the test material.<br>1 rat died due to food clogging (day 481).
POT (0.50 mg)	4	1 rat died during TIPS administration of the test material.<br>1 rat died from urethral obstruction (day 334).<br>1 rat died from a skin fibroma (day 350).<br>1 rat died from unknown causes (day 407).
MWCNT-7 (0.50 mg)	6	1 rat died from a brain tumor (day 304).<br>1 rat died from leukemia (day 463).<br>4 rats died due to food clogging (days 372, 414, 465, 484).

Table S3: Final body weights.

Group	Animals Surviving to week 105	Body weights at week 105
Untreated	15	448.3 ± 23.3
Vehicle	13	427.4 ± 25.4*
r-nTiO_2 (0.25 mg)	14	425.7 ± 24.5
r-nTiO2 (0.50 mg)	17	437.8 ± 55.5
POT (0.25 mg)	9	447.1 ± 23.7
POT (0.50 mg)	11	413.1 ± 36.1
MWCNT-7 (0.50 mg)	8	407.1 ± 25.8

* Different from the Vehicle Control at $p<0.05$.

Table S4: Neoplastic lesions found in individual rats.

Group	Animals with tumors	Lung Tumors	Pleural Mesotheiomas	Time of Death
Untreated	3	Bronchiolo-alveolar adenocarcinoma		TS (week 105)
		Bronchiolo-alveolar adenocarcinoma		TS (week 105)
		Bronchiolo-alveolar adenocarcinoma		TS (week 105)
Vehicle	1	Bronchiolo-alveolar adenoma		TS (week 105)
r-nTiO_2 (0.25 mg)	2	Bronchiolo-alveolar adenoma		TS (week 105)
		Bronchiolo-alveolar adenoma		TS (week 105)
r-nTiO2 (0.50 mg)	2	Bronchiolo-alveolar adenoma		TS (week 105)
		Bronchiolo-alveolar adenoma		TS (week 105)
POT (0.25 mg)	4	Bronchiolo-alveolar adenoma	Visceral mesothelioma	MS (week 94)
		Squamous cell carcinoma		TS (week 105)
			Parietal mesothelioma	MS (week 98)
			Parietal mesothelioma	TS (week 105)
POT (0.50 mg)	2	Bronchiolo-alveolar adenocarcinoma		TS (week 105)
		Bronchiolo-alveolar adenocarcinoma	Parietal mesothelioma	FD (week 75)
MWCNT-7 (0.50 mg)	7	Bronchiolo-alveolar adenoma		TS (week 105)
		Bronchiolo-alveolar adenocarcinoma		FD (week 73)
		Bronchiolo-alveolar adenocarcinoma		FD (week 92)
		Bronchiolo-alveolar adenocarcinoma		TS (week 105)
		Bronchiolo-alveolar adenocarcinoma		TS (week 105)
		Bronchiolo-alveolar adenocarcinoma	Parietal mesothelioma	MS (week 91)
			Parietal mesothelioma	FD (week 92)

TS: Terminal sacrifice
MS: Moribund sacrifice
FD: Found dead

Table S5: Incidence of other tumors.

Group	Animals examined	Leydig cell tumors	Leukemia/ Lymphoma	Subcutaneous fibroma	Preputial gland tumors	Pituitary tumors	Other
Untreated	19	10	1	2	5	0	Renal cortex adenoma (1)
Vehicle	19	11	1	3	1	4	
r-nTiO_2 (0.25 mg)	20	11	1	3	6	2	Oligodendroglioma (1)
r-nTiO2 (0.50 mg)	19	12	1	3	3	3	Undifferentiated pancreatic carcinoma (1)
POT (0.25 mg)	17	8	2	4	2	2	Renal cortex adenocarcinoma (1)<br>Undifferentiated pancreatic carcinoma (1)<br>Diaphragmatic carcinoma (1)<br>Nasal cavity squamous cell carcinoma (1)
POT (0.50 mg)	16	7	0	3	1	2	Undifferentiated pancreatic carcinoma (1)<br>Nasal cavity squamous cell carcinoma (2)
MWCNT-7 (0.50 mg)	14	8	2	2	2	2	Anaplastic Meningeal sarcoma (1)

Table S6: Absolute organ weights (grams).

Group	Animals examined	Lungs	Brain	Heart	Liver	Kidneys	Spleen
Untreated	15	1.36 ± 0.10	2.15 ± 0.03	1.16 ± 0.06	14.52 ± 0.85	2.99 ± 0.20	1.11 ± 0.36
Vehicle	13	1.30 ± 0.07	2.13 ± 0.06	1.12 ± 0.08	13.54 ± 1.42[#]	2.84 ± 0.23	0.94 ± 0.14
r-nTiO_2 (0.25 mg)	14	1.36 ± 0.08	2.15 ± 0.02	1.15 ± 0.10	13.97 ± 1.47	2.95 ± 0.19	1.11 ± 0.22
r-nTiO2 (0.50 mg)	17	1.34 ± 0.06	2.13 ± 0.04	1.16 ± 0.12	14.12 ± 1.70	3.00 ± 0.23	1.13 ± 0.36
POT (0.25 mg)	9	1.35 ± 0.07[a]	2.11 ± 0.05	1.15 ± 0.13	14.68 ± 1.97	3.05 ± 0.49[a]	1.13 ± 0.25
POT (0.50 mg)	11	1.35 ± 0.09	2.13 ± 0.03	1.10 ± 0.05	13.47 ± 2.11	2.83 ± 0.11	1.08 ± 0.44
MWCNT-7 (0.50 mg)	8	1.94 ± 0.73[*]	2.16 ± 0.04	1.16 ± 0.07	14.89 ± 2.05	2.99 ± 0.23	2.45 ± 2.84

* : Significantly different from vehicle group at $p < 0.05$.

# : Significantly different from untreated group at $p < 0.05$.

a : n=8, one organ was omitted.

Table S7: Relative organ weights (expressed as percent body weight).

Group	Animals examined	Lungs	Brain	Heart	Liver	Kidneys	Spleen
Untreated	15	0.31 ± 0.03	0.48 ± 0.03	0.26 ± 0.02	3.24 ± 0.18	0.67 ± 0.05	0.25 ± 0.09
Vehicle	13	0.31 ± 0.02	0.50 ± 0.03	0.26 ± 0.02	3.17 ± 0.31	0.67 ± 0.06	0.22 ± 0.03
r-nTiO_2 (0.25 mg)	14	0.32 ± 0.03	0.51 ± 0.03	0.27 ± 0.03	3.28 ± 0.31	0.69 ± 0.04	0.26 ± 0.06
r-nTiO2 (0.50 mg)	17	031 ± 0.03	0.49 ± 0.05	0.27 ± 0.02	3.25 ± 0.37	0.69 ± 0.08	0.26 ± 0.08
POT (0.25 mg)	9	0.30 ± 0.02[a]	0.47 ± 0.02	0.26 ± 0.03	3.28 ± 0.40	0.68 ± 0.11[a]	0.25 ± 0.06
POT (0.50 mg)	11	0.33 ± 0.04	0.52 ± 0.05	0.27 ± 0.02	3.27 ± 0.51	0.69 ± 0.07	0.26 ± 0.11
MWCNT-7 (0.50 mg)	8	0.48 ± 0.19*	0.53 ± 0.03*	0.28 ± 0.02*	3.66 ± 0.45**	0.74 ± 0.05*	0.61 ± 0.72

* : Significantly different from vehicle group at $p < 0.05$.

a : n=8, one organ was omitted.

Table S8. Non-neoplastic lesions in the lung and pleura.

Group	Untreated	Vehicle	r-nTiO_2 0.25 mg	r-nTiO_2 0.50 mg	POT 0.25 mg	POT 0.50 mg	MWCNT-7 0.50 mg
Number of animals	19	19	20	19	17	16	14
Alveolar type II epithelial cell hyperplasia	2	2	0	2	7	10**	14***
Bronchiolar hyperplasia	0	0	0	0	0	0	3
Alveolar macrophages[a]	0	0	0	0	4*	13***	14**
Focal fibrosis in the lung	0	0	0	0	4*	10***	14***
Granulamatous change in the lung	0	0	0	0	0	0	14***
Mesothelial cell hyperplasia	0	0	0	0	4*	7**	10***

[a] Alveolar macrophages were scored as +, ++, +++. The numbers of rats with scores of ++ and +++ are shown.

*,**,*** Different from both the Untreated and the Vehicle Control at $p<0.05$, $p<0.01$, $p<0.001$, respectively.

Table S9: LDH and Total Protein found in the pleural lavage fluid of individual rats.

Group	Tumor	LDH (U/L)	Total protein (µg/ml)
Untreated		5.12	292.79
		5.38	335.62
		8.00	351.13
		8.69	310.83
		9.46	359.14
Vehicle		4.90	430.76
		5.23	375.98
		7.39	266.13
		8.18	292.12
	Bronchiolo-alveolar adenoma	13.74	537.92
r-nTiO_2 (0.25 mg)		3.89	215.04
		5.10	373.28
		5.95	332.94
	Squamous cell carcinoma	11.33	475.65
	Mesothelioma (epitheliod)	14.78	3234.02
r-nTiO2 (0.50 mg)		5.22	275.45
		5.77	343.68
		6.21	331.60
		6.96	155.07
	Bronchiolo-alveolar adenocarcinoma	11.15	842.98
POT (0.25 mg)		4.37	66.26
		5.01	104.01
		5.14	914.28
		5.64	400.28
	Bronchiolo-alveolar adenoma	16.74	1112.18
POT (0.50 mg)		5.53	238.22
		6.18	351.07
		6.64	375.98
		6.76	409.07
		6.77	231.59
MWCNT-7 (0.50 mg)		5.96	324.89
		7.39	360.49
		7.50	321.54
		7.59	660.98
	Bronchiolo-alveolar adenocarcinoma	10.66	2726.85
