# Supplementary material for: Carcinogenic effect of potassium octatitanate (POT) fibers in the lung and pleura of male Fischer 344 rats after intrapulmonary administration
Source: Part Fibre Toxicol. 2019 Sep 2;16:34. doi: 10.1186/s12989-019-0316-2 (PMC6720102; doi:10.1186/s12989-019-0316-2)
Supplement: Supplementary file 4 — Survival curves, body weights, and fibers in the mediastinal lymph nodes of rats administered POT and MWCNT-7 fibers. Figure S1. Kaplan Meier survival curves. Figure S2. Body weight curves. Figure S3. Mediastinal lymph nodes of rats treated with POT and MWCNT-7 fibers. (PDF 5648 kb) [file 12989_2019_316_MOESM4_ESM.pdf]

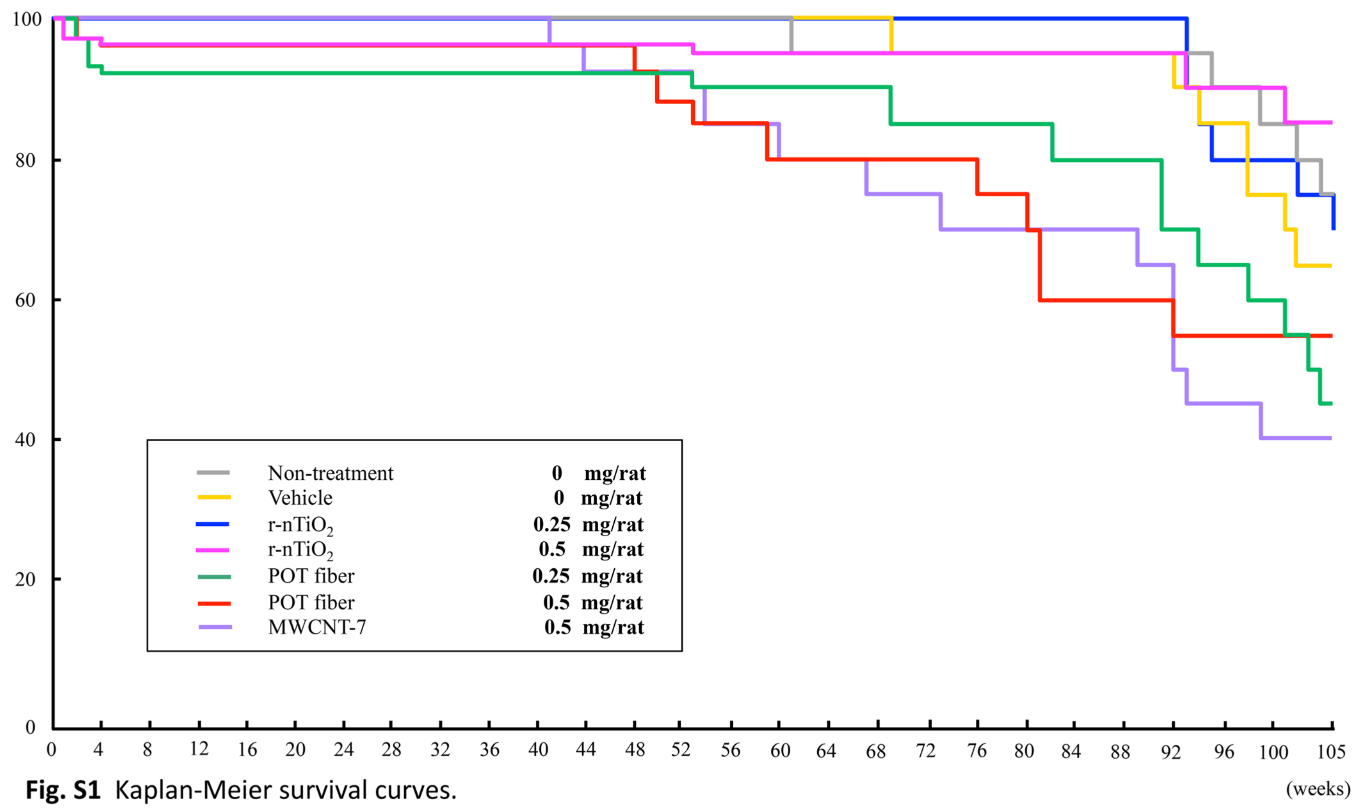


**Fig. S1** Kaplan-Meier survival curves.

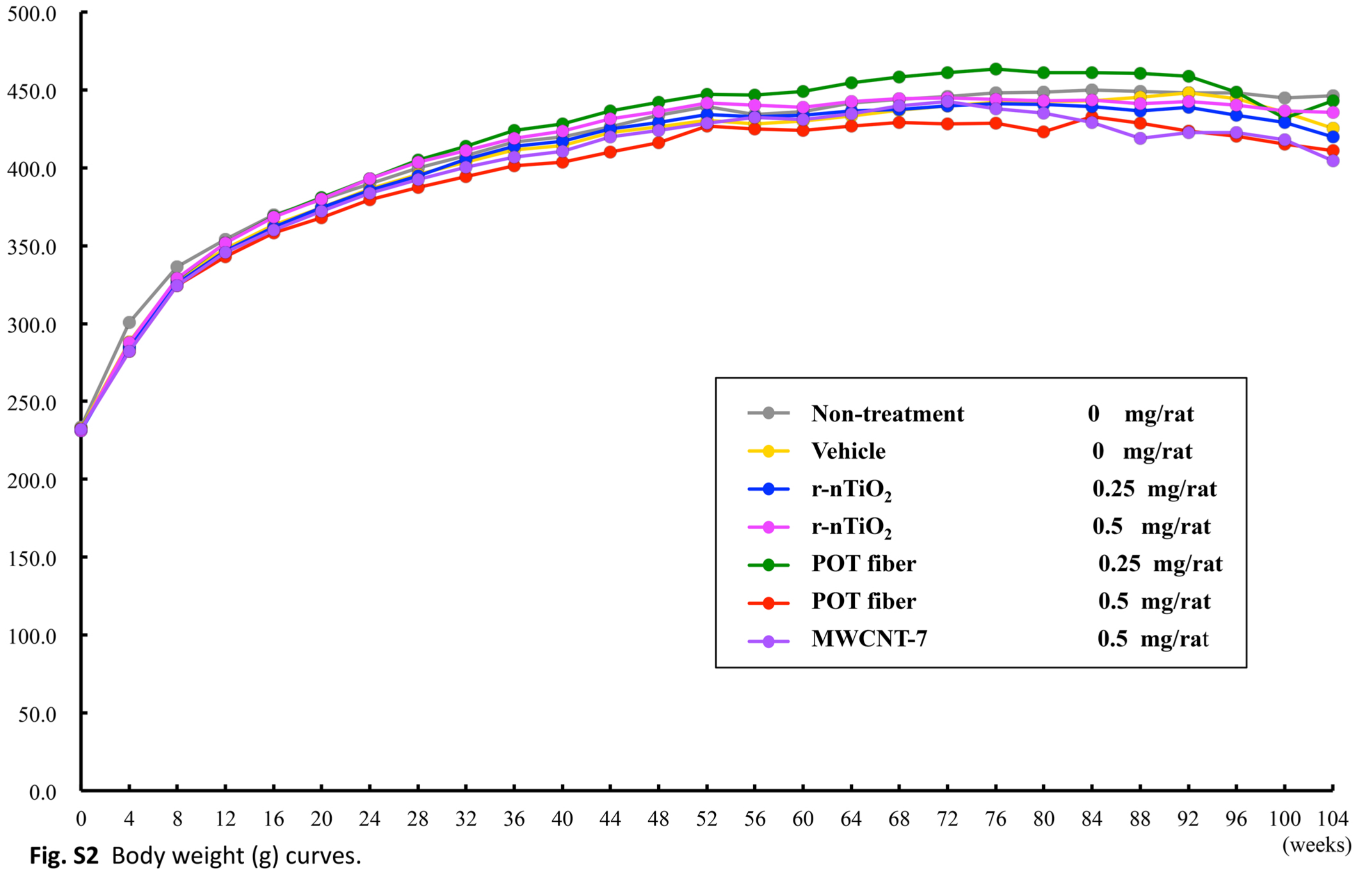


**Fig. S2** Body weight (g) curves.

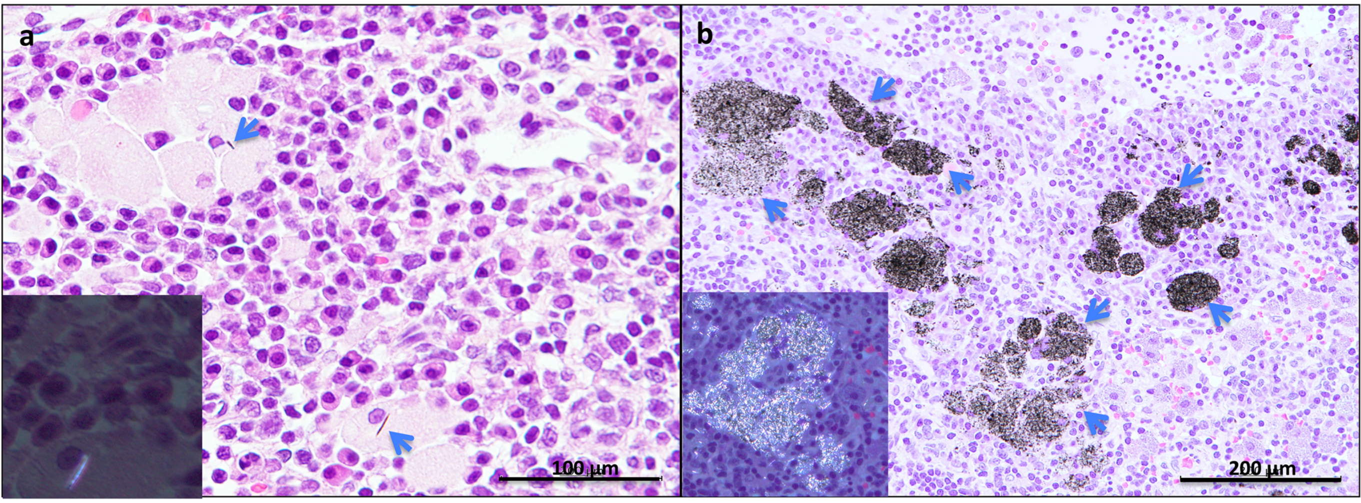


**Fig. S3** Mediastinal lymph nodes of rats treated with POT fibers (a) and MWCNT-7 (b).

**Figure S1.** Kaplen-Meier survival plots of untreated and vehicle control rats and rats treated with 0.25 mg r-n$TiO_2$, 0.50 mg r-n$TiO_2$, 0.25 mg POT fibers, 0.50 mg POT fibers, and 0.50 mg MWCNT-7. The MWCNT-7 treated rats had a significant reduction in survival rate (40%) compared to the vehicle control group ($p<0.05$).

**Figure S2.** Body weight curves of untreated and vehicle control rats and rats treated with 0.25 mg r-n$TiO_2$, 0.50 mg r-n$TiO_2$, 0.25 mg POT fibers, 0.50 mg POT fibers, and 0.50 mg MWCNT-7. There was no growth retardation in the rats administered r-n$TiO_2$, POT fibers, or 0.50 MWCNT-7 compared to the vehicle control group.

**Figure S3.** Mediastinal lymph nodes of rats treated with POT fibers (a) and MWCNT-7 (b). The amount of fibers (blue arrows) translocated into the mediastinal lymph nodes in MWCNT-7 treated rats was obviously higher than in the POT fiber treated rats. Insert, polarized light microscopic image.
