# Supplementary material for: Carcinogenic effect of potassium octatitanate (POT) fibers in the lung and pleura of male Fischer 344 rats after intrapulmonary administration
Source: Part Fibre Toxicol. 2019 Sep 2;16:34. doi: 10.1186/s12989-019-0316-2 (PMC6720102; doi:10.1186/s12989-019-0316-2)
Supplement: Supplementary file 5 — Lung Fiber Burdens in 2-year POT Studies. (PDF 176 kb) [file 12989_2019_316_MOESM5_ESM.pdf]

Lung Fiber Burdens in 2-yr POT Studies

Our current study is the only study that instilled POT fibers into the lungs of test animals and followed the animals for 2 years. Four studies exposed animals to POT fibers by inhalation and followed the exposed animals for up to 2 years (Table 1).

We instilled 0.5 mg POT fibers into the lungs of 10-week old male F344 rats. These fibers had a mean length of 6.06 ± 1.53 µm and mean width of 305 ± 69 nm. The density of the fibers was 3.5 g/cm^3. Thus, 0.5 mg of this material contained 3.23 x 10^8 fibers. Given an absolute wet lung weight of 1 g and dry weight of 22% of the wet weight [1], the rats were exposed to approximately 1500 fibers/µg dry weight lung tissue.

Ikegami et al. (2004) exposed male F344 rats to up to up to 200 POT fibers/cm^3 for 6 h/day, 5 days/wk for 24 months [22]. At the end of the 2-yr exposure period the lung burden was 254.2 x 10^6 WHO fibers per lung in the high exposure group. The lung weight was 2.224 ± 0.165 g in this group. Assuming a dry weight of 22% of the wet weight, the fiber burden in these rats was 520 fibers/µg dry weight lung tissue.

Yamato et al. (2003) exposed male Wistar rats to 2.2 mg/m^3 (111 fibers/cm^3) POT fibers for 6 h/day, 5 days/wk for 1 yr and followed the animals for up to 1 year after the end of the exposure period [2]. At the end of the 1-yr exposure period the lung burden was 2.36 ± 0.72 mg/lung [3]. Assuming a wet lung weight of 1.5 g in the 1-yr old rats and a dry weight of 22% of the wet weight, the fiber burden in these rats was 361 fibers/µg dry weight lung tissue: this also assumes that nasal filtering did not change the fiber number/weight ratio of the fibers deposited in the lung

Oyabu et al. (2004) exposed male Wistar rats to 1.9 mg/m^3 POT fibers for 6 h/day, 5 days/wk for 1 yr and followed the animals for up to 1 year after the end of the exposure period [3]. At the end of the 1-yr exposure period the lung burden was 2.39

± 0.50 mg/lung [3]. The number of fibers/unit weight was not stated in the Oyabu manuscript.

Lee et al. (1981) exposed rats, hamsters, and guinea pigs to up to 371 mg/m^3 POT fibers (101,500 fibers/cm^3) for 6h/day, 5 days/wk for 3 months and followed the animals for an additional 15 - 24 months (fibers longer than 5 μM and less than 10 μM were approximately 35-45% of the total fibers) [4]. Lung burdens were not stated in the Lee manuscript.

Table 1. Summary of Two-Year Inhalation Studies

Fiber Concentration (mg/m^3)	Fiber Number (fibers/cm^3)	
79<br>39<br>82<br>371	8,530<br>6,720<br>36,100<br>101,500	Lee 1981
2.2	111	Yamato 2003
1.9	Not Stated	Oyabu 2004
Not Stated	Up to 200	Ikegami 2004

References

1. Tillery SI, Lehnert BE. Age-bodyweight relationships to lung growth in the F344 rat as indexed by lung weight measurements. Lab Anim. 1986;20 3:189-94; doi: 10.1258/002367786780865610. https://www.ncbi.nlm.nih.gov/pubmed/3795855.
2. Yamato H, Oyabu T, Ogami A, Morimoto Y, Higashi T, Tanaka I, et al. Pulmonary effects and clearance after long-term inhalation of potassium octatitanate whiskers in rats. Inhal Toxicol. 2003;15 14:1421-34; doi: 10.1080/08958370390248969. http://www.ncbi.nlm.nih.gov/pubmed/14648357.
3. Oyabu T, Yamato H, Ogami A, Morimoto Y, Akiyama I, Ishimatsu S, et al. The effect of lung burden on biopersistence and pulmonary effects in rats exposed to potassium octatitanate whiskers by inhalation. J Occup Health. 2004;46 5:382-90. http://www.ncbi.nlm.nih.gov/pubmed/15492455.

4. Lee KP, Barras CE, Griffith FD, Waritz RS. Pulmonary response and transmigration of inorganic fibers by inhalation exposure. Am J Pathol. 1981;102 3:314-23. https://www.ncbi.nlm.nih.gov/pubmed/7212016.
