# Supplementary material for: Carcinogenic effect of potassium octatitanate (POT) fibers in the lung and pleura of male Fischer 344 rats after intrapulmonary administration
Source: Part Fibre Toxicol. 2019 Sep 2;16:34. doi: 10.1186/s12989-019-0316-2 (PMC6720102; doi:10.1186/s12989-019-0316-2)
Supplement: Supplementary file 6 — Lessons from Malignant Pleural Mesothelioma. (PDF 319 kb) [file 12989_2019_316_MOESM6_ESM.pdf]

Lessons from Malignant Pleural Mesothelioma

Malignant mesothelioma occurs as a result of neoplastic transformation of mesothelial cells lining the pleural, peritoneal, or pericardial cavities [1]. Malignant pleural mesothelioma is generally associated with exposure to fibrous mineral particles such as asbestos and erionite [1, 2]. Asbestos fibers interact directly with the tissues in which they are deposited, causing generation of oxidants, chromosomal damage, and activation of cell signaling pathways [3, 4, 5, 6, 7, 8].

Asbestos-mediated activation of signaling pathways that promote cellular replication has been postulated to contribute to asbestos-associated malignancies. However, it is highly unlikely that this contributes to the initial stages of asbestos-associated neoplastic transformation. Multiple extracellular antiproliferative signals operate within normal tissues to maintain tissue homeostasis, and in addition, there are numerous intracellular pathways that operate to prevent inappropriate cell replication in response to aberrant proliferative signaling. These checkpoint-pathways include the LKB1, NF-2, and Rb associated pathways, oncogene-induced replicative stress pathways, and DNA damage response pathways, and lead to suppression of cellular proliferation, irreversible senescence, or apoptosis or necrosis [9, 10, 11, 12, 13, 14, 15, 16]. Accordingly, before a normal human cell can undergo anomalous proliferation, it must acquire mutations that allow it to bypass the tissue and cellular checkpoints that inhibit deregulated DNA replication and cellular proliferation.

It has also been proposed that chromosomal damage induced by direct interaction of asbestos with the DNA or by interference of asbestos with mitosis or cytokinesis contributes to the development of asbestos-associated malignancies. However, it is highly unlikely that this type of DNA damage contributes to the initial stages of asbestos-associated neoplastic transformation because, unlike checkpoint-

compromised cells, overt chromosomal damage such as chromosome breakage or asymmetric chromosome segregation will cause normal checkpoint-intact cells to undergo senescence, apoptosis, or necrosis [9, 10, 12, 13]. For example, one well documented pathway activated by asbestos is induction of p53 expression resulting in senescence or apoptosis of cells with irreparable DNA damage [5, 17, 18, 19]. Thus, before gross chromosomal damage can lead to carcinogenesis-associated mutations, checkpoints that monitor such damage and block proliferation of damaged cells, either by senescence or cell death pathways, must be bypassed. Taken altogether, the points noted above argue that the source of the initial asbestos-mediated DNA damage that leads to malignant pleural mesothelioma is the generally minor damage caused by reactive oxygen (ROS) and nitrogen (RNS) species generated by asbestos interactions with tissue cells and phagocytes and ROS generated by iron complexed with asbestos fibers via iron catalyzed Fenton reactions [6, 7], and that the initial mutations that lead to asbestos-induced neoplastic transformation occur in checkpoint genes.

DNA mutations require 2 indispensable elements: (1) DNA damage and (2) mis-repair of the damage or mis-replication of the damaged DNA during cellular proliferation in order to fix the mutation into the genome. Mis-repair of oxidative DNA damage (as argued above, in checkpoint-intact cells more extensive DNA damage will result in senescence or cell death) results in mismatched bases. Mismatched bases are repaired by the mismatch repair system, which is found throughout the prokaryotic and eukaryotic kingdoms. This system is generally associated with repair of mismatched bases generated during DNA replication, but when mismatched bases are recognized outside the context of DNA replication, discrimination between the correct base and the mismatched base is lost, and repair of

the mismatch can lead to fixation of a mutation into the genome [20]. Mis-replication of an oxidatively damaged DNA base occurs when the base pairs with a non-cognate DNA base during DNA replication [21]: oxidatively damaged DNA bases can also pair with cognate DNA bases or they can lead to cytotoxicity, in which cases a mutation does not occur [21].

Most DNA damage is caused by cell endogenous agents or is the result of an error occurring during DNA replication or mitosis [22]. The rate of DNA damage per cell per day is not precisely known, however, cells are estimated to have approximately 1,000 - 10,000 8-oxo-dG adducts [23] in addition to myriad other types of damage. Therefore, the rate of DNA damage in normal cells is significant. However, repair of DNA lesions or induction of senescence or the death of cells harboring damaged DNA is highly efficient. This can be demonstrated by the extremely low incidence of malignant pleural mesotheliomas that develop spontaneously in humans, approximately equal to or less than 1 to 2 per one million people [1, 24]. The low incidence of spontaneous malignant pleural mesothelioma development indicates that mis-repair of damaged DNA in a pleural mesothelial cell, fixation of the consequent mutation in the cell, and proliferation of the mutation bearing cell will occur very infrequently during a person's lifetime. In addition, since the pleural mesothelium is a renewing tissue with approximately 0.16 to 0.5% of the cells undergoing mitosis at any one time [25], the incidence of spontaneous malignant pleural mesothelioma argues that damaged DNA is repaired before it is replicated. Of course, mutations will accrue in pleural mesothelial cells, but the fidelity of DNA repair mechanisms coupled with the checkpoints that block carcinogenesis[9, 10] effectively prevent spontaneous cancer development in the pleural mesothelium.

In spite of the robust DNA monitoring and repair pathways operating in human cells, asbestos-mediated oxidative DNA damage can result in mutations. But, since oxidative DNA damage is recognized and effectively repaired by the DNA repair systems of human cells, how can asbestos-mediated oxidative DNA damage cause mutations that eventually lead to development of malignant pleural mesothelioma? One possibility is that asbestos-mediated DNA damage overwhelms the capacity of the cell's DNA monitoring and repairs systems. However, this is unlikely as the reported asbestos fiber burden of pleural mesothelioma tissue is generally lower than the number of fibers required to increase mutation frequency in rats and mice. Topinka et al., 2004, [26] administered 1 mg, 2 mg, or 4 weekly doses of 2 mg amosite asbestos by intratracheal instillation into the lungs of male transgenic λ-LacI Big Blue™ rats. They examined the rats for mutations 4 wk and 16 wk after the last treatment and found that 1 mg amosite asbestos did not increase mutation frequency above background levels, i.e., above the number of mutations that had accumulated in the rat tissue since embryogenesis, at either the 4 wk or 16 wk time points. Assuming a fiber weight of 7.4×10^5 fibers/μg ([878 fibers/0.0296 mm^2] x [1 mm^2/0.04 μg]) [27] and a lung dry weight of 0.33 g [28] the lung fiber burden of rats administered 1 mg amosite asbestos would be 2.2×10^9 fibers/g lung tissue. In another mutagenesis study using male transgenic LacI Big Blue™ mice, Rihn et al., 2000, [29] administered crocidolite asbestos by nose-only inhalation 6hr/day for 5 consecutive days and examined the mice for mutations 1 wk, 4 wk, and 12 wk after the last exposure. Mutation frequency was not increased at 1 wk but was significantly increased at 4 wk; however, at 12 wk the mutation frequency was at background levels, suggesting that mutation bearing cells had been mostly eliminated. The lung fiber burden in these mice was 1.63×10^9 fibers/g lung tissue (dry weight) at 1 wk and

0.62 x 10^9 fibers/g at 12 wk. Suzuki and Yuen, 2001, [30] examined asbestos fiber burdens in human pleural mesothelioma tissues. They found that fiber burden ranged from 0.03 x 10^6 fibers/g dry tumor tissue to 240 x 10^6 fibers/g with a median of 17 x 10^6 fibers/g and an average of 49.84 x 10^6 fibers/g. In a later study of 168 pleural mesothelioma cases Suzuki et al, 2004, [31] found an average fiber burden of 46.5 x 10^6 fibers/g dry tumor tissue. In an earlier study of pleural fiber burden of asbestos-exposed patients, Boutin et al., 1996, [32] found that in the parietal pleura (the primary site of asbestos fiber deposition and pleural mesothelioma development [7, 33]) normal tissue contained 0.5 x 10^6 fibers/g dry tissue while the area around the parietal pleura stomata, which are sites of lymphatic drainage and accumulate particles that have been deposited in the pleural cavity [33, 34], contained 4.1 x 10^6 fibers/g dry tissue. Thus, assuming a 10-fold increase in fiber burden in certain areas of the mesothelioma tissues examined by Suzuki and co-workers, a fiber burden of approximately 500 x 10^6 fibers/g could be anticipated, which is still generally lower than the fiber burden required to increase mutation frequency in rats and mice. Importantly, DNA maintenance and repair systems are much more robust in long-lived humans than in short-lived species such as mice [35, 36], indicating that much higher levels of DNA damage are required to overwhelm the DNA monitoring and repair systems of human cells and increase mutation frequency compared to rats and mice. Thus, while assessment of asbestos fiber burden is uncertain [34], and the proportion of active fibers and inactive sequestered fibers present during the development of mesothelioma in these patients is unknown, the reported data of fiber burden in pleural mesothelioma tissue support the premise that fiber burdens below those that are overtly mutagenic can be carcinogenic and argue that asbestos-mediated DNA damage does not necessarily overwhelm the DNA monitoring and repair

systems of pleural mesothelial cells. Another point is that people can develop pleural mesothelioma after low level occupational or non-occupational exposure to asbestos, which results in lower but still carcinogenic fiber burdens [37, 38], further supporting the premise that fiber burdens below those that are overtly mutagenic can be carcinogenic.

A simple proposal that can explain the ability of low, seemingly non-mutagenic levels of asbestos fibers to cause malignant carcinomas is that these fibers cause damage to the DNA of proliferating cells. Thus, even if only a very low level of DNA damage is generated, the damage may not be repaired prior to DNA replication and could result in mutations in proliferating cells: mutations acquired by a relatively few proliferating cells may not be discernable when assessing the mutation frequency of whole tissue, but could nevertheless eventually lead to neoplastic transformation.

The arguments presented above assert that during the initial stages of asbestos-associated neoplastic transformation, the DNA monitoring and repair systems of pleural mesothelial cells are not overwhelmed by asbestos-engendered DNA damage, but rather proliferating cells acquire non-lethal oxidative DNA damage and that this damaged DNA is replicated before it is repaired. Three properties of asbestos contribute to this process: 1) asbestos can directly damage the tissue; 2) asbestos induces an inflammatory response; 3) asbestos induces the generation of reactive oxygen species (ROS) and reactive nitrogen species (RNS) [3, 4, 5, 6, 7, 8].

Asbestos can induce necrosis or apoptosis of all relevant lung and pleural cells, especially mesothelial cells [8, 39]. Asbestos-mediated induction of cell death brings about a tissue repair response and consequent proliferation of potential target cells.

Asbestos induces inflammatory responses via multiple routes. Asbestos can induce necrosis of mesothelial cells [8, 39], and cells undergoing necrosis release intracellular proteins such as HMGB1 (high mobility group box 1) that act as danger associated molecular patterns (DAMPs), also known as alarmins, that interact with and activate inflammatory cells [40, 41, 42]. Interaction of asbestos with living tissue cells can also promote inflammatory signaling [3, 4, 43]. In addition, it is well known that interaction of asbestos with phagocytes elicits an inflammatory response [6]. Mediators released during the inflammatory response can cause tissue damage, bringing about a tissue repair response and consequent proliferation of potential target cells [44].

Like asbestos-associated inflammatory responses, asbestos induces generation of ROS and RNS species by multiple routes. ROS can be generated by iron complexed with asbestos fibers via iron catalyzed Fenton reactions [7]. Interaction of asbestos with tissue cells can also induce the generation of ROS and RNS [3, 4], which can damage the DNA of neighboring cells even if the asbestos interacting cell dies. Interaction of asbestos fibers with phagocytes also results in the generation of ROS and RNS [6, 45, 46, 47, 48]. Therefore, asbestos-associated tissue damage coupled with asbestos-associated generation of ROS and RNS establish conditions that enable these reactive oxidants to damage the DNA of proliferating cells.

A cell must acquire multiple mutations before it is able to undergo deregulated replication; therefore, multiple cycles of tissue damage, tissue repair, and damage of the DNA of replicating cells must occur in order for a cell to acquire the requisite mutations for neoplastic transformation. In addition to acquiring DNA damage and fixing consequent mutations into daughter cells, cycles of replication also increase the number of target cells. This is essential, as all the mutations required for

carcinogenesis must accumulate in one cell. As noted above, asbestos itself and asbestos-associated inflammatory responses engender such cycles. If exposure to asbestos fibers is terminated, the cycles of tissue damage/repair will also cease and the neoplastic transformation process will arrest. Since mutations caused by asbestos exposure are random, the exact number of tissue damage/repair cycles an asbestos-exposed cell undergoes before it is able to undergo deregulated replication is not predictable, but it commonly takes decades before normal mesothelial cells exposed to asbestos acquire the mutations that allow them to generate a detectable tumor [1, 2, 49, 50].

One of the consequences of the random nature of mutagenesis coupled with the high fidelity of a human cell's DNA maintenance and repair systems is that it has been argued that the requisite mutations needed for neoplastic transformation are highly unlikely to occur within a human lifetime, leading to the possibility that some early mutations negatively impact DNA maintenance and repair [51]. This mutator phenotype has been described as an enabling characteristic of cancer [9, 10].

Once a cell has acquired the requisite mutations that allow deregulated replication, it is able to respond to mutations that promote its proliferation. During the promotion phase of tumorigenesis, the rate of replication, and therefore the rate of mutation, will increase. Asbestos-associated damage and inflammatory responses also play a key role in the later stages of carcinogenesis as the initiated cells acquire additional mutations, which can include the gross chromosomal damage known to be caused by asbestos, that ultimately result in the formation of an invasive tumor with the capability to metastasize [10, 52, 53, 54, 55, 56, 57, 58, 59, 60, 61, 62] (also see ref [63] and references therein).

Therefore, we propose the following points: (1) To acquire the initial mutations required for neoplastic transformation, asbestos-engendered reactive oxidants must damage the DNA of proliferating cells. (2) Asbestos-engendered reactive oxidants are capable of damaging the DNA of proliferating cells because (a) asbestos causes tissue damage, both directly and through asbestos-induced inflammatory responses, and this results in cell proliferation to repair the damage, and (b) asbestos causes the generation of DNA damaging oxidants. Thus, the asbestos-engendered oxidants are able to damage the DNA of the cells proliferating in response to asbestos-associated tissue damage. (3) Asbestos fibers do not need to cause a high level of DNA-damage since the damage is in proliferating cells; consequently, even low, incidental exposure to asbestos can cause damage to DNA that is replicated before it is repaired, thereby enabling mutations to be fixed into the genomes of daughter cells. (4) The initial mutations that enable cells to begin to undergo neoplastic transformation target genes that allow the cell to bypass tissue and cellular checkpoints that inhibit deregulated DNA replication, and perhaps mutations that target the cell's DNA maintenance and repair systems to allow the cell to acquire a mutator phenotype. (5) During this initial stage, the cell will proliferate in a normal and regulated manner, and consequently, repeated cycles of asbestos-associated tissue damage and repair are required for the initiation of asbestos-associated carcinogenesis. The idea that asbestos-associated inflammatory responses and repeated cycles of asbestos-associated tissue damage and repair are required for the development of malignant pleural mesothelioma is more than a decade old. Coussens and Warb, 2002, writing about inflammation and cancer (with pleural mesothelioma being associated with asbestos-induced inflammation): "Hence, repeated tissue damage and regeneration of tissue, in the presence of highly reactive nitrogen and oxygen species released from inflammatory cells, interacts with

DNA in proliferating epithelium resulting in permanent genomic alterations such as point mutations, deletions, or rearrangements." [61] Okada and Fujii, 2006, also writing about inflammation and cancer (with pleural mesothelioma being associated with asbestos-induced inflammation): "It is assumed, therefore, that continuous generation of ROS/RNS by phagocytes may injure cells. This could, in turn, cause compensatory cell proliferation, which will effectively accumulate DNA damage and gene mutations; all these steps are essential to carcinogenesis." [64] Topinka et al., 2004, wrote "We propose that asbestos-induced mutations develop gradually from oxidative DNA lesions being converted into mutations in proliferating cells during a continuous inflammation process." [26] Yang et al., 2008, wrote "In particular, crocidolite is generally considered to be the most oncogenic type of asbestos. The long and thin fibers (especially $\geq$ 8 µm in length $\leq$ 0.25 µm in width) are thought to be more dangerous, because they have longer biopersistance in the pleura. These fibers are able to penetrate the lung and cause repeated damage, tissue repair and local inflammation." [8]

Concluding Remarks:

The fidelity of the DNA maintenance and repair systems of mammalian cells is exceedingly effective. Therefore, repair of non-lethal asbestos-associated DNA damage is very efficient. Consequently, unless a pleural mesothelial cell sustains levels of damage that overwhelm the cell's DNA maintenance and repair systems, a resting normal pleural mesothelial cell is highly unlikely to acquire mutations caused by asbestos-associated DNA damage. Since the pleural mesothelium is not exposed to levels of asbestos fibers that would cause enough DNA damage to overwhelm the cell's DNA maintenance and repair systems, we propose that oxidative damage of the

DNA of actively propagating cells is essential for the generation of the initial asbestos-induced mutations that eventually lead to the development of malignant pleural mesothelioma. This proposition requires that DNA-damaging oxidants are generated in the immediate vicinity of proliferating cells. Since asbestos fibers cause both oxidant generation and tissue damage followed by tissue repair, exposure of the pleural mesothelium to asbestos results in proliferating cells being exposed to DNA-damaging oxidants. The DNA damaging oxidants are ROS and RNS species generated when asbestos interacts with mesothelial cells and phagocytes, and ROS can also be generated by iron complexed with asbestos fibers via iron catalyzed Fenton reactions. Asbestos fibers cause tissue damage by direct interaction with tissue cells and also indirectly via asbestos-associated inflammatory responses. Proximate mesothelial cells proliferate in response to this damage and are consequently exposed to asbestos-engendered DNA-damaging oxidants. After numerous cycles of asbestos-associated tissue damage/repair, a few cells will accumulate the mutations required to bypass many of the cell's innate proliferative and cellular damage checkpoints, initiating the neoplastic transformation process. Ultimately, as mutations accumulate and the cells' interaction with the host progresses, pre-neoplastic lesions develop and some of these lesions can harbor cells capable of evolving into cancers of the pleural mesothelium.

Importantly, this proposal argues that asbestos-mediated generation of mutations will begin upon exposure of the tissue to asbestos fibers and does not require asbestos fibers to accumulate to exceedingly high levels before generation of DNA mutations begins. But continued exposure to asbestos fibers, enabled by biopersistence and repeated exposure to asbestos fibers and opposed by the body's defenses, is required for the multiple cycles of tissue damage and repair in the

presence of DNA damaging oxidants that is required for initiation and perpetuation of the carcinogenic process. The higher the level of exposure to asbestos fibers, the more the body's defenses against inhaled particles will be frustrated, and the inflammatory responses, generation of DNA damaging oxidants, and tissue damage will become stronger and more extensive. This will result in DNA damage and mutations in an increasing number of proliferating cells, augmenting the carcinogenic process.

These arguments also apply to other inhaled particles. We propose that any particle that causes inflammatory responses and generation of reactive oxidants and multiple cycles of tissue damage and tissue repair is a potential carcinogen.

References

1. Carbone M, Ly BH, Dodson RF, Pagano I, Morris PT, Dogan UA, et al. Malignant mesothelioma: facts, myths, and hypotheses. J Cell Physiol. 2012;227 1:44-58; doi: 10.1002/jcp.22724. http://www.ncbi.nlm.nih.gov/pubmed/21412769.

2. U.S. Public Health Service. U.S. Department of Health and Human Services. Toxicological profile for asbestos. Atlanta, GA: Agency for Toxic Substances and Disease Registry; 2001. 2001. http://www.atsdr.cdc.gov/toxprofiles/tp61.pdf. https://www.atsdr.cdc.gov/ToxProfiles/tp.asp?id=30&tid=4

3. Nymark P, Wikman H, Hienonen-Kempas T, Anttila S. Molecular and genetic changes in asbestos-related lung cancer. Cancer Lett. 2008;265 1:1-15; doi: 10.1016/j.canlet.2008.02.043. http://www.ncbi.nlm.nih.gov/pubmed/18364247.

4. Huang SX, Partridge MA, Ghandhi SA, Davidson MM, Amundson SA, Hei TK. Mitochondria-derived reactive intermediate species mediate asbestos-induced genotoxicity and oxidative stress-responsive signaling pathways. Environ Health Perspect. 2012;120 6:840-7; doi: 10.1289/ehp.1104287. http://www.ncbi.nlm.nih.gov/pubmed/22398240.

5. Huang SX, Jaurand MC, Kamp DW, Whysner J, Hei TK. Role of mutagenicity in asbestos fiber-induced carcinogenicity and other diseases. J Toxicol Environ Health B Crit Rev. 2011;14 1-4:179-245; doi: 10.1080/10937404.2011.556051. http://www.ncbi.nlm.nih.gov/pubmed/21534089.

6. IARC, WHO. Asbestos (chrysotile, amosite, crocidolite, tremolite, actinolite, and anthophyllite). IARC Monographs on the Evaluation of Carcinogenic Risks to Humans. A Review of Human Carcinogens; 100 C: Arsenic, Metals, Fibres, and Dusts. Lyon, France, 2012; 219–309. 2012. http://monographs.iarc.fr/ENG/Monographs/vol100C/mono100C.pdf.

7. Chew SH, Toyokuni S. Malignant mesothelioma as an oxidative stress-induced cancer: An update. Free Radic Biol Med. 2015;86:166-78; doi: 10.1016/j.freeradbiomed.2015.05.002. http://www.ncbi.nlm.nih.gov/pubmed/25975982.

8. Yang H, Testa JR, Carbone M. Mesothelioma epidemiology, carcinogenesis, and pathogenesis. Curr Treat Options Oncol. 2008;9 2-3:147-57; doi: 10.1007/s11864-008-0067-z. http://www.ncbi.nlm.nih.gov/pubmed/18709470.

9. Hanahan D, Weinberg RA. The hallmarks of cancer. Cell. 2000;100 1:57-70. http://www.ncbi.nlm.nih.gov/pubmed/10647931.

10. Hanahan D, Weinberg RA. Hallmarks of cancer: the next generation. Cell. 2011;144 5:646-74; doi: 10.1016/j.cell.2011.02.013.

http://www.ncbi.nlm.nih.gov/pubmed/21376230.

11. Vaahtomeri K, Makela TP. Molecular mechanisms of tumor suppression by LKB1. FEBS Lett. 2011;585 7:944-51; doi: 10.1016/j.febslet.2010.12.034. https://www.ncbi.nlm.nih.gov/pubmed/21192934.

12. Lowe SW, Cepero E, Evan G. Intrinsic tumour suppression. Nature. 2004;432 7015:307-15; doi: 10.1038/nature03098. https://www.ncbi.nlm.nih.gov/pubmed/15549092.

13. Fridman JS, Lowe SW. Control of apoptosis by p53. Oncogene. 2003;22 56:9030-40; doi: 10.1038/sj.onc.1207116. https://www.ncbi.nlm.nih.gov/pubmed/14663481.

14. Gan RY, Li HB. Recent progress on liver kinase B1 (LKB1): expression, regulation, downstream signaling and cancer suppressive function. Int J Mol Sci. 2014;15 9:16698-718; doi: 10.3390/ijms150916698. https://www.ncbi.nlm.nih.gov/pubmed/25244018.

15. Okada T, Lopez-Lago M, Giancotti FG. Merlin/NF-2 mediates contact inhibition of growth by suppressing recruitment of Rac to the plasma membrane. J Cell Biol. 2005;171 2:361-71; doi: 10.1083/jcb.200503165. https://www.ncbi.nlm.nih.gov/pubmed/16247032.

16. Curto M, Cole BK, Lallemand D, Liu CH, McClatchey AI. Contact-dependent inhibition of EGFR signaling by Nf2/Merlin. J Cell Biol. 2007;177 5:893-903; doi: 10.1083/jcb.200703010. https://www.ncbi.nlm.nih.gov/pubmed/17548515.

17. Kamp DW. Asbestos-induced lung diseases: an update. Transl Res. 2009;153 4:143-52; doi: 10.1016/j.trsl.2009.01.004. https://www.ncbi.nlm.nih.gov/pubmed/19304273.

18. Liu G, Cheresh P, Kamp DW. Molecular basis of asbestos-induced lung disease. Annu Rev Pathol. 2013;8:161-87; doi: 10.1146/annurev-pathol-020712-163942. https://www.ncbi.nlm.nih.gov/pubmed/23347351.

19. Panduri V, Surapureddi S, Soberanes S, Weitzman SA, Chandel N, Kamp DW. P53 mediates amosite asbestos-induced alveolar epithelial cell mitochondria-regulated apoptosis. Am J Respir Cell Mol Biol. 2006;34 4:443-52; doi: 10.1165/rcmb.2005-0352OC. https://www.ncbi.nlm.nih.gov/pubmed/16357363.

20. Crouse GF. Non-canonical actions of mismatch repair. DNA Repair (Amst). 2016;38:102-9; doi: 10.1016/j.dnarep.2015.11.020. https://www.ncbi.nlm.nih.gov/pubmed/26698648.

21. Dizdaroglu M. Oxidatively induced DNA damage and its repair in cancer. Mutat Res Rev Mutat Res. 2015;763:212-45; doi: 10.1016/j.mrrev.2014.11.002.

http://www.ncbi.nlm.nih.gov/pubmed/25795122.

22. Chatterjee N, Walker GC. Mechanisms of DNA damage, repair, and mutagenesis. Environ Mol Mutagen. 2017;58 5:235-63; doi: 10.1002/em.22087. https://www.ncbi.nlm.nih.gov/pubmed/28485537.

23. Collins AR. Oxidative DNA damage, antioxidants, and cancer. Bioessays. 1999;21 3:238-46; doi: 10.1002/(SICI)1521-1878(199903)21:3<238::AID-BIES8>3.0.CO;2-3. https://www.ncbi.nlm.nih.gov/pubmed/10333733.

24. Lemen RA. Mesothelioma from asbestos exposures: Epidemiologic patterns and impact in the United States. J Toxicol Environ Health B Crit Rev. 2016;19 5-6:250-65; doi: 10.1080/10937404.2016.1195323. https://www.ncbi.nlm.nih.gov/pubmed/27705549.

25. Mutsaers SE. The mesothelial cell. Int J Biochem Cell Biol. 2004;36 1:9-16. https://www.ncbi.nlm.nih.gov/pubmed/14592528.

26. Topinka J, Loli P, Georgiadis P, Dusinska M, Hurbankova M, Kovacikova Z, et al. Mutagenesis by asbestos in the lung of lambda-lacI transgenic rats. Mutat Res. 2004;553 1-2:67-78; doi: 10.1016/j.mrfmmm.2004.06.023. https://www.ncbi.nlm.nih.gov/pubmed/15288534.

27. Lowers HA, Bern AM. Particle size characterization of water-elutriated Libby amphibole 2000 and RTI international amosite: U.S. Geological Survey Open-File Report #1242. Available at: http://pubs.usgs.gov/of/2009/1242/downloads. 2009.

28. Tillery SI, Lehnert BE. Age-bodyweight relationships to lung growth in the F344 rat as indexed by lung weight measurements. Lab Anim. 1986;20 3:189-94; doi: 10.1258/002367786780865610. https://www.ncbi.nlm.nih.gov/pubmed/3795855.

29. Rihn B, Coulais C, Kauffer E, Bottin MC, Martin P, Yvon F, et al. Inhaled crocidolite mutagenicity in lung DNA. Environ Health Perspect. 2000;108 4:341-6. https://www.ncbi.nlm.nih.gov/pubmed/10753093.

30. Suzuki Y, Yuen SR. Asbestos tissue burden study on human malignant mesothelioma. Ind Health. 2001;39 2:150-60. https://www.ncbi.nlm.nih.gov/pubmed/11341545.

31. Suzuki Y, Yuen SR, Ashley R. Asbestos Fiber Analysis in the Lung and Mesothelial Tissues from 168 Cases of Human Malignant Mesothelioma. Global Asbestos Congress (GAC 2004). Available at: http://worldasbestosreport.org/conferences/gac/gac2004/pl_4_01_e.pdf. 2004.

32. Boutin C, Dumortier P, Rey F, Viallat JR, De Vuyst P. Black spots concentrate oncogenic asbestos fibers in the parietal pleura. Thoracoscopic and mineralogic study. Am J Respir Crit Care Med.

1996;153 1:444-9; doi: 10.1164/ajrccm.153.1.8542156. https://www.ncbi.nlm.nih.gov/pubmed/8542156.

33. Donaldson K, Murphy FA, Duffin R, Poland CA. Asbestos, carbon nanotubes and the pleural mesothelium: a review of the hypothesis regarding the role of long fibre retention in the parietal pleura, inflammation and mesothelioma. Part Fibre Toxicol. 2010;7:5; doi: 10.1186/1743-8977-7-5. https://www.ncbi.nlm.nih.gov/pubmed/20307263.

34. Broaddus VC, Everitt JI, Black B, Kane AB. Non-neoplastic and neoplastic pleural endpoints following fiber exposure. J Toxicol Environ Health B Crit Rev. 2011;14 1-4:153-78; doi: 10.1080/10937404.2011.556049. http://www.ncbi.nlm.nih.gov/pubmed/21534088.

35. MacRae SL, Croken MM, Calder RB, Aliper A, Milholland B, White RR, et al. DNA repair in species with extreme lifespan differences. Aging (Albany NY). 2015;7 12:1171-84; doi: 10.18632/aging.100866. https://www.ncbi.nlm.nih.gov/pubmed/26729707.

36. MacRae SL, Zhang Q, Lemetre C, Seim I, Calder RB, Hoeijmakers J, et al. Comparative analysis of genome maintenance genes in naked mole rat, mouse, and human. Aging Cell. 2015;14 2:288-91; doi: 10.1111/acel.12314. https://www.ncbi.nlm.nih.gov/pubmed/25645816.

37. Wolff H, Vehmas T, Oksa P, Rantanen J, Vainio H. Asbestos, asbestosis, and cancer, the Helsinki criteria for diagnosis and attribution 2014: recommendations. Scand J Work Environ Health. 2015;41 1:5-15; doi: 10.5271/sjweh.3462. http://www.ncbi.nlm.nih.gov/pubmed/25299403.

38. Gilham C, Rake C, Burdett G, Nicholson AG, Davison L, Franchini A, et al. Pleural mesothelioma and lung cancer risks in relation to occupational history and asbestos lung burden. Occup Environ Med. 2016;73 5:290-9; doi: 10.1136/oemed-2015-103074. https://www.ncbi.nlm.nih.gov/pubmed/26715106.

39. Napolitano A, Antoine DJ, Pellegrini L, Baumann F, Pagano I, Pastorino S, et al. HMGB1 and Its Hyperacetylated Isoform are Sensitive and Specific Serum Biomarkers to Detect Asbestos Exposure and to Identify Mesothelioma Patients. Clin Cancer Res. 2016;22 12:3087-96; doi: 10.1158/1078-0432.CCR-15-1130. http://www.ncbi.nlm.nih.gov/pubmed/26733616.

40. Bianchi ME. DAMPs, PAMPs and alarmins: all we need to know about danger. J Leukoc Biol. 2007;81 1:1-5; doi: 10.1189/jlb.0306164. http://www.ncbi.nlm.nih.gov/pubmed/17032697.

41. Piccinini AM, Midwood KS. DAMPening inflammation by modulating TLR signalling. Mediators Inflamm. 2010;2010; doi: 10.1155/2010/672395.

http://www.ncbi.nlm.nih.gov/pubmed/20706656.

42. Harris HE, Andersson U, Pisetsky DS. HMGB1: a multifunctional alarmin driving autoimmune and inflammatory disease. Nat Rev Rheumatol. 2012;8 4:195-202; doi: 10.1038/nrrheum.2011.222. http://www.ncbi.nlm.nih.gov/pubmed/22293756.

43. Luster MI, Simeonova PP. Asbestos induces inflammatory cytokines in the lung through redox sensitive transcription factors. Toxicol Lett. 1998;102-103:271-5. https://www.ncbi.nlm.nih.gov/pubmed/10022265.

44. Chovatiya R, Medzhitov R. Stress, inflammation, and defense of homeostasis. Mol Cell. 2014;54 2:281-8; doi: 10.1016/j.molcel.2014.03.030. http://www.ncbi.nlm.nih.gov/pubmed/24766892.

45. Donaldson K, Brown GM, Brown DM, Bolton RE, Davis JM. Inflammation generating potential of long and short fibre amosite asbestos samples. Br J Ind Med. 1989;46 4:271-6. http://www.ncbi.nlm.nih.gov/pubmed/2540793.

46. Kamp DW, Weitzman SA. The molecular basis of asbestos induced lung injury. Thorax. 1999;54 7:638-52. http://www.ncbi.nlm.nih.gov/pubmed/10377212.

47. Vallyathan V, Shi X, Castranova V. Reactive oxygen species: their relation to pneumoconiosis and carcinogenesis. Environ Health Perspect. 1998;106 Suppl 5:1151-5. http://www.ncbi.nlm.nih.gov/pubmed/9788890.

48. Matsuzaki H, Maeda M, Lee S, Nishimura Y, Kumagai-Takei N, Hayashi H, et al. Asbestos-induced cellular and molecular alteration of immunocompetent cells and their relationship with chronic inflammation and carcinogenesis. J Biomed Biotechnol. 2012;2012:492608; doi: 10.1155/2012/492608. http://www.ncbi.nlm.nih.gov/pubmed/22500091.

49. Bianchi C, Giarelli L, Grandi G, Brollo A, Ramani L, Zuch C. Latency periods in asbestos-related mesothelioma of the pleura. Eur J Cancer Prev. 1997;6 2:162-6. http://www.ncbi.nlm.nih.gov/pubmed/9237066.

50. Nadler DL, Zurbenko IG. Estimating cancer latency times using a Weibull model. Advances in Epidemiology. 2014;2014.

51. Loeb LA. Mutator phenotype may be required for multistage carcinogenesis. Cancer Res. 1991;51 12:3075-9. http://www.ncbi.nlm.nih.gov/pubmed/2039987.

52. Mantovani A. Cancer: Inflaming metastasis. Nature. 2009;457 7225:36-7; doi: 10.1038/457036b. http://www.ncbi.nlm.nih.gov/pubmed/19122629.

53. Colotta F, Allavena P, Sica A, Garlanda C, Mantovani A. Cancer-

related inflammation, the seventh hallmark of cancer: links to genetic instability. Carcinogenesis. 2009;30 7:1073-81; doi: 10.1093/carcin/bgp127. http://www.ncbi.nlm.nih.gov/pubmed/19468060.

54. Balkwill F, Charles KA, Mantovani A. Smoldering and polarized inflammation in the initiation and promotion of malignant disease. Cancer Cell. 2005;7 3:211-7; doi: 10.1016/j.ccr.2005.02.013. http://www.ncbi.nlm.nih.gov/pubmed/15766659.

55. Ben-Neriah Y, Karin M. Inflammation meets cancer, with NF-kappaB as the matchmaker. Nat Immunol. 2011;12 8:715-23; doi: 10.1038/ni.2060. http://www.ncbi.nlm.nih.gov/pubmed/21772280.

56. Medzhitov R. Origin and physiological roles of inflammation. Nature. 2008;454 7203:428-35; doi: 10.1038/nature07201. http://www.ncbi.nlm.nih.gov/pubmed/18650913.

57. Pribluda A, Elyada E, Wiener Z, Hamza H, Goldstein RE, Biton M, et al. A senescence-inflammatory switch from cancer-inhibitory to cancer-promoting mechanism. Cancer Cell. 2013;24 2:242-56; doi: 10.1016/j.ccr.2013.06.005. http://www.ncbi.nlm.nih.gov/pubmed/23890787.

58. Rybinski B, Franco-Barraza J, Cukierman E. The wound healing, chronic fibrosis, and cancer progression triad. Physiol Genomics. 2014;46 7:223-44; doi: 10.1152/physiolgenomics.00158.2013. http://www.ncbi.nlm.nih.gov/pubmed/24520152.

59. Grivennikov SI, Greten FR, Karin M. Immunity, inflammation, and cancer. Cell. 2010;140 6:883-99; doi: 10.1016/j.cell.2010.01.025. http://www.ncbi.nlm.nih.gov/pubmed/20303878.

60. Rakoff-Nahoum S. Why cancer and inflammation? Yale J Biol Med. 2006;79 3-4:123-30. https://www.ncbi.nlm.nih.gov/pubmed/17940622.

61. Coussens LM, Werb Z. Inflammation and cancer. Nature. 2002;420 6917:860-7; doi: 10.1038/nature01322. https://www.ncbi.nlm.nih.gov/pubmed/12490959.

62. Balkwill F, Mantovani A. Inflammation and cancer: back to Virchow? Lancet. 2001;357 9255:539-45; doi: 10.1016/S0140-6736(00)04046-0. https://www.ncbi.nlm.nih.gov/pubmed/11229684.

63. Mantovani A. The inflammation - cancer connection. FEBS J. 2018;285 4:638-40; doi: 10.1111/febs.14395. https://www.ncbi.nlm.nih.gov/pubmed/29479848.

64. Okada F, Fujii J. Molecular mechanisms of inflammation-induced carcinogenesis. Journal of Clinical Biochemistry and Nutrition. 2006;39 3:103-13.
